# A Novel HURRAH Protocol Reveals High Numbers of Monomorphic MHC Class II Loci and Two Asymmetric Multi-Locus Haplotypes in the Père David's Deer

**DOI:** 10.1371/journal.pone.0014518

**Published:** 2011-01-18

**Authors:** Qiu-Hong Wan, Pei Zhang, Xiao-Wei Ni, Hai-Long Wu, Yi-Yan Chen, Ye-Ye Kuang, Yun-Fa Ge, Sheng-Guo Fang

**Affiliations:** 1 The Key Laboratory of Conservation Biology for Endangered Wildlife of the Ministry of Education, College of Life Sciences, State Conservation Center for Gene Resources of Endangered Wildlife, Zhejiang University, Hangzhou, People's Republic of China; 2 College of Life Science, Anhui Normal University, Wuhu, People's Republic of China; American Museum of Natural History, United States of America

## Abstract

The Père David's deer is a highly inbred, but recovered, species, making it interesting to consider their adaptive molecular evolution from an immunological perspective. Prior to this study, genomic sequencing was the only method for isolating all functional MHC genes within a certain species. Here, we report a novel protocol for isolating MHC class II loci from a species, and its use to investigate the adaptive evolution of this endangered deer at the level of multi-locus haplotypes. This protocol was designated “HURRAH” based on its various steps and used to estimate the total number of MHC class II loci. We confirmed the validity of this novel protocol in the giant panda and then used it to examine the Père David's deer. Our results revealed that the Père David's deer possesses nine MHC class II loci and therefore has more functional MHC class II loci than the eight genome-sequenced mammals for which full MHC data are currently available. This could potentially account at least in part for the strong survival ability of this species in the face of severe bottlenecking. The results from the HURRAH protocol also revealed that: (1) All of the identified MHC class II loci were monomorphic at their antigen-binding regions, although DRA was dimorphic at its cytoplasmic tail; and (2) these genes constituted two asymmetric functional MHC class II multi-locus haplotypes: DRA1*01 ∼ DRB1 ∼ DRB3 ∼ DQA1 ∼ DQB2 (H1) and DRA1*02 ∼ DRB2 ∼ DRB4 ∼ DQA2 ∼ DQB1 (H2). The latter finding indicates that the current members of the deer species have lost the powerful ancestral MHC class II haplotypes of nine or more loci, and have instead fixed two relatively weak haplotypes containing five genes. As a result, the Père David's deer are currently at risk for increased susceptibility to infectious pathogens.

## Introduction

The major histocompatibility complex (MHC) is a highly polymorphic region composed of three tightly linked genomic regions that are termed class I, class II, and class III. The MHC class II genes encode cell-surface glycopeptides that present bacterial-derived antigens to CD4+ T cells, thereby triggering immune responses [1]. The exon 2 domain of MHC is responsible for the recognition of diverse pathogens, and comprises the most polymorphic functional marker found in vertebrates [2]. The functional class II molecule is a heterodimer consisting of an alpha gene-encoded α chain and a beta gene-encoded β chain [3]. In mammals, the MHC class II cluster has developed from ancestral paired alpha and beta genes into three genomic sub-regions (DR, DQ, and DP); these regions include numerous classical antigen-presenting loci, including the DRAs, DRBs, DQAs, DQBs, DPAs, and DPBs [4]–[5]. A multitude of MHC class II genes occupy a genomic region spanning several hundred kilobases in length [4]–[10], making it very difficult for researchers to resolve all of the MHC genes in a given species using polymerase chain reaction (PCR)-based techniques.

However, in view of the pivotal roles played by MHC genes, scientists have developed elaborate methods for exploring these genes. Such methods typically involve three key sequential steps: the construction of a genomic library; physical mapping of the MHC genes; and isolation/identification of the MHC genes through genomic sequencing and functional analysis in different species. Because such procedures are complicated and costly, they have been used for only a few commonly studied animals. Some examples of the MHC gene clusters that have been fully elucidated to date are the mouse (H2), human (HLA), cat (FLA), dog (DLA), pig (SLA), cow (BoLA), and sheep (*Ovar-*MHC) [4]–[10]. However, wild or semi-wild animals are considered to be better models for studying adaptive survival under pathogenic conditions [11]–[13] and behavioral mechanisms in natural populations [14]–[16]. Unfortunately, such work requires a great deal of data on the MHC genes and their genomic sequences, and this information has not yet been fully elucidated in wild animals. Previous MHC studies in wild animals have typically used cross-species primers originally derived from domestic animal sequences [16]–[20]. However, the results from such studies may be biased or even misleading, due to the relative lack of explored loci, the potential for the identification of pseudogenes, the risk of cross-locus amplification, and other issues. Although cross-species primer amplification has allowed the identification of numerous cDNA sequences corresponding to MHC genes, few of the previous studies have successfully identified defined loci (Table 1). Therefore, it would clearly be useful to replace the expensive and time-consuming three-step procedures with a novel method capable of isolating all MHC loci from wild animals or other rarely studied species.

**Table 1:** Species distribution and locus identification of 148 MHC class II cDNA sequences of wild carnivores and ungulates obtained from NCBI.

Taxonomic group	Species involved	Number of sequences	Gene categories involved	Objective of cDNA isolation	Number of loci defined successfully	References/GenBank No.
Cervidae	*Cervus elaphus*	69	DRB and DQB	RACE-based locus isolation	Undefined: up to two DRBs; more than one DQB	[27], [47]
Delphinidae	*Tursiops truncatus*	10	DRB and DQB	PCR-based locus isolation	Undefined: both at least two DRBs and one DQB	[48]
	*Tursiops aduncus*	10	DRB and DQB	PCR-based locus isolation		
	*Cephalorhynchus hectori*	3	DQB	Check expression	/	[49]
Balaenidae	*Eubalaena australis*	4	DQB	Check expression	/	[50]
Pontoporiidae	*Pontoporia blainvillei*	1	DQB	Check expression	/	[50]
Bovidae	*Bubalus bubalis*	8	DRA, DRB, DQA and DQB	PCR-based locus isolation	Two DQAs	[51]
	*Capra hircus*	3	DRA and DRB	Check expression	/	[52]
	*Ovibos moschatus*	1	DRB	/	/	AF387317
	*Hemitragus jemlahicus*	1	DRB	/	/	AF336341
	*Rupicapra rupicapra*	1	DRB	PCR-based locus isolation	One expressed DRB and one pseudogene	[53]
Hippopotamidae	*Hippopotamus amphibius*	2	DRB and DQB	/	/	EF017819- 20
Otariidae	*Zalophus californianus*	34	DRA, DRB, DQA and DQB	RACE-based locus isolation	Undefined: all multiple	[30], [54]
Canidae	*Alopex lagopus*	1	DQA	Check expression	/	[55]

The Père David's deer (*Elaphurus davidianus*; Chinese name: milu) is listed as extinct in the wild in the IUCN (International Union for Conservation of Nature) 2010 Red List (http://www.iucnredlist.org). This deer originally lived in northeastern and east-central China, but became extinct in the late 19th century [21]. At present, all of the Père David's deer in existence worldwide are descended from 11 of 18 individuals that were kept at Woburn Abbey in England during 1894∼1904 [22]. Despite the fact that there were only 11 ancestral founders, however, the Père David's deer has successfully passed through the genetic bottleneck of inbreeding, and has well adapted to the vast open parklands of the English country estate where they have been housed since 1894 [23]. Furthermore, the strong survival ability of the Père David's deer has allowed these animals to repeatedly overcome bottlenecks when building new breeding populations.

In China, the first reintroduction of the Père David's deer into Beijing Nanhaizi Milu Park was initiated by a group of 38 founders obtained from Woburn Abbey in 1985 (20 heads) and 1987 (18 heads). The second reintroduction into Dafeng Milu National Nature Reserve were performed by a group of 39 and 19 deer collected from seven London zoos in 1986 and Whipsnad Wild Animal Park in 1987, respectively. Subsequently, descendant deer were exported from the Beijing population to Shishou in 1993, Yuanyang in 2003, and Linan in 2005 with the aim of building new breeding groups. By 2003, the deer species had successfully expanded to a total of about 3,000 individuals distributed across 156 breeding sites in 23 countries of Asia, Africa, Europe, America, and Oceania [22]. Despite its highly inbred genetic background, this deer species has shown no obvious evidence of inbreeding depression [24], and its members have adapted well to different environments on five continents. Thus, it could be interesting to explore the adaptive molecular evolution of this species from the perspective of its MHC genetics. However, the first step in conducting a detailed characterization of the MHC genes for this rare species will be the isolation of all expressed MHC loci and such work is constrained by the above-described limitations.

Here, we describe our newly developed method for isolating functional MHC class II genes without the use of a genomic library or physical mapping. We report the validation of this method utilizing the giant panda genome resources available within our laboratory, and then describe our use of the new technique to isolate MHC class II genes from the Père David's deer. Finally, we report the characterization of the genetic variations at all MHC class II genes in two semi-wild Père David's deer populations, as analyzed using single-strand conformation polymorphism (SSCP), heteroduplex (HD), and sequence analyses. Our results provide the first molecular explanation for the high-level adaptive ability of the inbred, but recovered, Père David's deer.

## Results

### Overview of the new protocol

**Figure 1:**
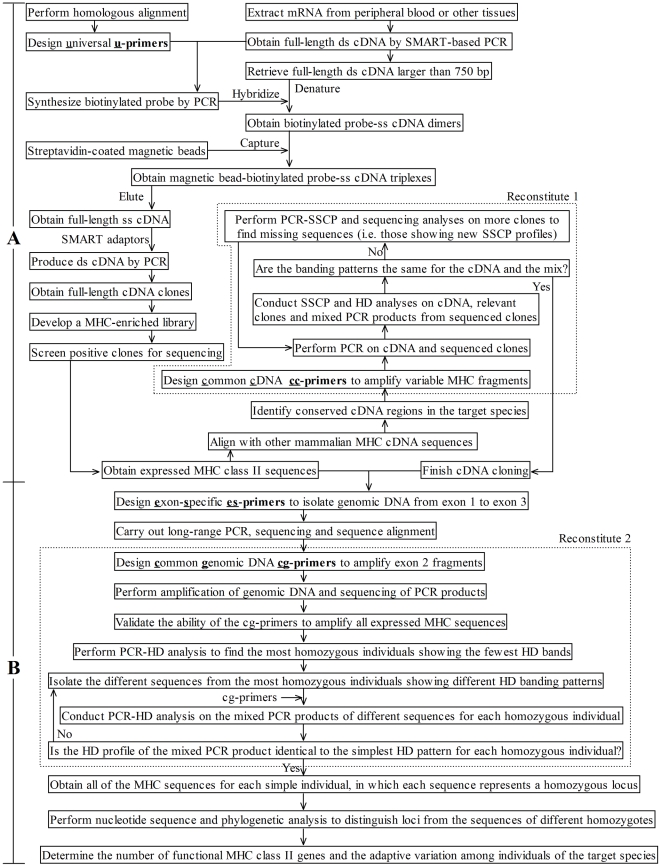
Technical design of the novel HURRAH protocol for isolating MHC class II loci. Here we show only profile reconstitution of the simplest SSCP-HD banding patterns, i.e. those representing a homozygous haplotype. For practical use, the most complicate SSCP-HD profile should also be reconstituted for comparison.

The novel protocol presented herein consists of two parts: the isolation of expressed MHC sequences (HUR; Figure 1A), and the enumeration of the functional MHC class II loci (RAH; Figure 1B). The first part includes cDNA synthesis, universal probe preparation, magnetic bead-based hybridization, sequencing of random clones, and reconstitution of the SSCP-HD banding patterns, which is how we check that all of the expressed MHC sequences have been successfully isolated (Figure 1A). We first designed universal (u-series; Supplementary Table S1) primers for DRA, DRB, DQA, and DQB to synthesize PCR-based biotinylated probes. We hybridized the biotinylated probes to the denatured single-stranded (ss) cDNAs generated by the SMART (switching mechanism at 5′ end of RNA transcript) PCR, and thereby isolated the expressed MHC sequences. Next, we used streptavidin-coated magnetic beads to bind the biotinylated probe-ss cDNA dimers, thereby enriching our samples for the MHC class II sequences (Figure 1A). We then amplified the eluted ss cDNA sequences into double-stranded (ds) cDNA fragments, constructed a small MHC-enriched cDNA library, and randomly selected positive cDNA clones for sequencing. Finally, we designed common cDNA primers (cc-series; Supplementary Table S2) for the target species and utilized PCR-SSCP and HD to check for banding patterns that could help us identify any sequences that might have been missed (Figure 1A). More specifically, we amplified the inserts of the cDNA clones and mixed the amplified products to obtain their compound SSCP and HD banding patterns, and then compared these patterns with those obtained from PCR products amplified from the original cDNA. If the mixed and original SSCP and HD profiles were identical, we could conclude that all of the expressed MHC sequences had been successfully isolated. This part of the protocol had two main features: (1) magnetic bead-based cDNA **h**ybridization using biotinylated probes synthesized by PCR of **u**niversal primers; and (2) the use of profile **r**econstitution to ensure that we had isolated all of the desired cDNA sequences. Accordingly, we called the first part of the protocol “HUR.”

The second part of the protocol involved isolating the genomic DNA sequences that corresponded to the identified cDNA sequences, scanning for the most homozygous individuals, identifying the number of expressed MHC class II genes, and investigating the adaptive variations among individuals of the target species (Figure 1B). We first designed exon-specific primers (es-series; Supplementary Table S3) and used long-range (LR) PCR to isolate the genomic introns flanking each cDNA sequence. Next, we performed multiple sequence alignment of the intronic sequences and designed common genomic primers (cg-series; Supplementary Table S2) that amplified the variable antigen-presenting region. The cg-series primers were then employed to scan various individuals in search of those carrying the most homozygous MHC haplotypes (i.e., the fewest HD bands). For each of the simplest HD banding patterns, we utilized the cg-series primers to isolate the MHC sequences from a representative individual and mixed the PCR products from each isolated sequence to reconstitute the SSCP and HD banding patterns of original genomic PCR products. If all of the relevant MHC sequences had been isolated in the prior steps, we were able to identify all of the MHC sequences represented by the homozygous haplotype. Furthermore, each isolated sequence could be taken as representing a homozygous locus in that individual. This part of the protocol was characterized by one main feature: **r**econstituting the SSCP and HD banding patterns of the most homozygous individuals revealed by **a**nalysis of cg-primer **H**Ds, in order to ensure that we had isolated all of the sequences represented by the homozygous haplotypes. Therefore, we called the second part of the protocol “RAH,” and the two parts together, comprising our novel protocol, were designated “HURRAH.”

### Probe preparation and positive clone rate

The universal primers amplified products from both the carnivorous giant panda and the herbivorous Père David's deer (Figure S1). Agarose gel electrophoresis of the PCR products indicated successful biotinylation of the probes, which ran slower than the control PCR products (Figure S1). After hybridization, ligation, and transformation, we used amplification of u-series universal primers and subsequent sequencing to identify the positive clones. We found that the positive-clone rate varied from 14% (DRA) to 20% (DRB) in the giant panda and from 10% (DRA) to 95% (DRB) in the Père David's deer. We then constructed small MHC-enriched cDNA libraries for both the giant panda and the Père David's deer.

### Verification of the novel protocol using the giant panda

The results we obtained from the giant panda validated the ability of the HUR methods to isolate classical MHC class II loci from mRNA and the ability of the RAH approaches to identify multi-locus haplotype homozygotes (Supplementary data S1). Furthermore, the use of these new methods clarified a previous mistake, wherein the lack of the full-length cDNA sequence had led to the identification of two functional DRB genes instead of only one. Based on these findings, we next applied the novel HURRAH protocol to another mammal for which no MHC information was available: the Père David's deer.

### Identification of *Elaphurus davidianus* MHC (*Elda*-MHC) loci using the HURRAH protocol

#### Isolation of *Elda*-MHC class II cDNA and genomic sequences

We used the HURRAH protocol to successfully isolate ten different MHC sequences from the blood-derived mRNA of a Père David's deer (Figure S2); these sequences included two DRA sequences (named DRAa and DRAb), four DRB sequences (DRBa, DRBb, DRBc and DRBd), two DQA sequences (DQAa and DQAb), and two DQB sequences (DQBa and DQBb). Using the isolated sequences, we could successfully reconstitute the SSCP-HD banding patterns of the PCR products from the original cDNA sample (Figure S2), suggesting that we had isolated all of the expressed MHC sequences. Using es-series primers (Table S3), we obtained genomic DNA sequences spanning exons 1 through 3 for each MHC cDNA sequence.

#### Enumeration of the functional *Elda*-MHC class II loci

We used the cg-series (Table S2) primers to examine the genetic variations of exon 2 in two semi-wild populations. Our results suggested that the four gene categories each presented three types of SSCP-HD banding patterns: two simple ones and one relatively complex one (Figure S3). Sequence analysis revealed that the simpler profiles of DRA, DQA and DQB corresponded to single sequences (DRA: DRAa or DRAb; DQA: DQAa or DQAb; DQB: DQBa or DQBb), while the two simpler banding patterns of DRB were composed of DRBb+DRBd or DRBa+DRBc, respectively (Figure S3). The relatively complex profiles of these genes presented DRAa and DRAb for DRA; DRBa, DRBb, DRBc and DRBd for DRB; DQAa and DQAb for DQA; DQBa and DQBb for DQB (Figure S3). Obviously, the simpler patterns represented homozygotes, while the more complicated patterns corresponded to heterozygotes; this was verified by mixing the homozygotes to successfully reconstitute the heterozygotes. Given the mixed sequence compositions and the multi-band HD patterns obtained for the DRB homozygous haplotypes, we chose to reconstitute the three types of DRB SSCP-HD banding patterns. Profile reconstitution demonstrated that the three DRB banding patterns corresponded to DRBb+DRBd, DRBa+DRBc and DRBa+DRBb+DRBc+DRBd (Figure S4), indicating that we had successfully isolated all of the cDNA and genomic DNA sequences corresponding to the MHC genes in this species.

We then further analyzed the long MHC class II haplotypes involving four gene categories by locus linkage disequilibrium analysis. Interestingly, we found that individuals were either homozygous at all genes or heterozygous at all genes (Figure S3). Thus, our analysis identified two *Elda-*MHC class II haplotypes: DRAa ∼ DRBa ∼ DRBc ∼ DQAa ∼ DQBb (H1) and DRAb ∼ DRBb ∼ DRBd ∼ DQAb ∼ DQBa (H2). Animals could be homozygous for H1, homozygous for H2, or they could be H1/H2 heterozygotes. This finding was supported by multi-locus haplotype analysis using the PyPop software package [25]. Therefore, we obtained one DRA, two DRB, one DQA and one DQB genes for each *Elda*-MHC class II haplotype.

### Construction of *Elda*-MHC class II multi-locus haplotypes

#### Characteristics of nucleotide and amino acid sequences

**Figure 2:**
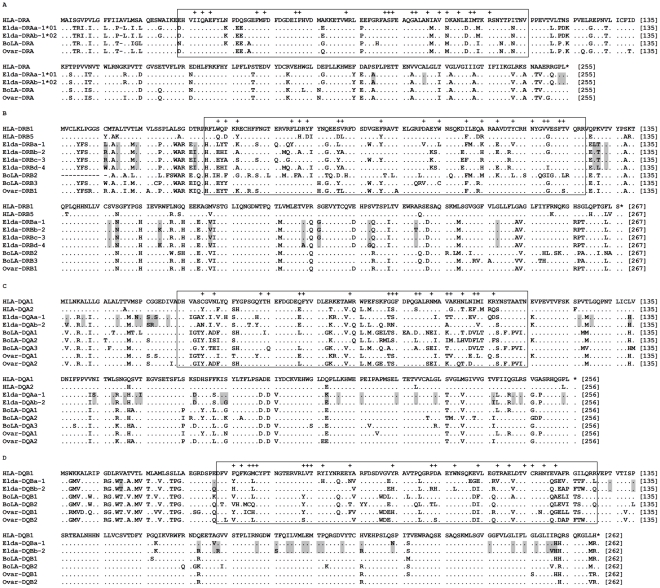
Multiple sequence alignments of the amino acid sequences deduced from the full-length cDNAs. Dots indicate identity to the first sequence and gaps represent missing amino acids. The box indicates antigen-presenting exon 2, and crosses indicate putative antigen-binding sites, as determined based on the HLA equivalents [42]. The letters and numbers following the *Elda*-MHC genes indicate their corresponding cDNA sequences, and the names of loci and alleles identified by the HURRAH protocol. The shaded areas indicate nucleotide differences among the *Elda*-MHC loci.

The nucleotide and deduced amino acid sequence alignments of the *Elda-*MHC sequences revealed the following: (1) All of the encoded genes had normal start and stop codons (Figure 2), and had the characteristics of classical MHC class II genes. (2) DRAa and DRAb were highly similar, with identical nucleotide sequences in their signal peptide, antigen-presenting and trans-membrane regions, and only three nucleotide differences (no amino acid differences) at the ends of their cytoplasmic tails (Figure 2). (3) Unlike DRA, the DRB, DQA, and DQB sequences possessed numerous specific nucleotide and amino acid variations in addition to those in their antigen-presenting regions. These variations were found within relatively invariable parts of each locus, including the signal peptide, trans-membrane and cytoplasmic regions (Figure 2), giving these sequences the genetic basis for becoming separate genes. Consistent with the above findings, the es-series primers produced intronic fragments of similar lengths for the DRAs, but differently sized intronic fragments for the DRBs, DQAs, and DQBs (Table S3).

#### Phylogenetic analysis

**Figure 3:**
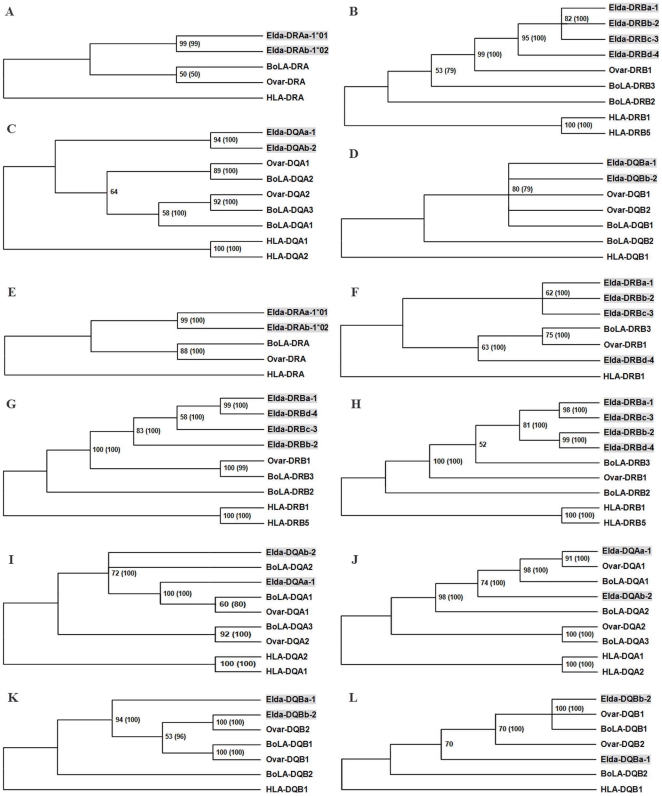
Phylogenetic trees for the DRA, DRB, DQA, and DQB loci. The trees were generated based on exon 3-exon 5 cDNA sequences (A, B, C, and D), intron 1 sequences (G, I, and K), intron 2 sequences (E, H, J and L), and 5′UTR-exon 1 (F). The complete intron 1 sequence of *Ovar*-DRA was unavailable, so we show only those based on the intron 2 sequences. The *Elda*-MHC genes are shaded, to allow them to be easily distinguished from the cow and sheep genes. Numbers indicate bootstrap percentages (values smaller than 50% are not shown). The numbers outside and inside the parentheses are bootstrap values for the MP and Bayesian trees, respectively. The bootstrap percentages of the NJ trees were very similar to those of the MP trees, and thus are not shown. As the branch lengths differed among trees, only the topologies are shown here. The information near each *Elda*-MHC gene indicates the initial cDNA sequence (a, b, c and d) and identified loci (1, 2, 3 and 4). For this analysis, the expressed HLA, BoLA and *Ovar*-MHC loci were identified from the relevant completely sequenced MHC class II regions of the human (HLA; NT_007592), cow (BoLA; Btau 3.1) and sheep (*Ovar*-MHC; EU176819).

We constructed neighbor-joining (NJ), maximum-parsimony (MP), and Bayesian phylogenetic trees for the trans-membrane and cytoplasmic regions (exon 3 through exon 5), introns 1 and −2, and the 5′-UTR through exon 1 in order to confirm the expected topologies containing one, two, one, and one paired branches for the DRAs, DRBs, DQAs and DQBs, respectively. In the exon 3-exon 5-based trees (Figure 3), the DRAs and DQAs formed confident branches in the Bayesian, NJ and MP trees, showing higher bootstrap values (>90%). The DRBs and DQBs had no highly-supportive pairing in all kinds of trees, showing that there were large differences among DRB and DQB sequences. Except for DQBs, all the MHC sequences of each gene category clustered together, reflecting an evolutionary history specific to this deer species. The two DQBs were grouped together with the sheep and cow DQBs with about 80% bootstrap values, providing evidence for the locus identities of the DQBa and DQBb.

The intron 2-based phylogenetic trees for DRA were consistent with those based on the exon 3-exon 5 sequences (99% supportive of a DRAa-DRBb pairing; Figure 3E), confirming that the *Elda*-DRAs corresponded to a single dimorphic DRA locus; accordingly, we renamed DRAa and DRAb as DRA1*01 and DRA1*02. In contrast to the consistent findings for the DRAs, the intron-based trees for the DQAs and DQBs were largely discordant (Figure 3). For the DQAs, the intron 1- and intron 2-based trees indicated that the closest relatives of *Elda*-DQAa were the cow BoLA-DQA1 and the sheep *Ovar*-DQA1; these pairings had high bootstrap values (98–100%; Figures 3I and 3J), indicating that *Elda*-DQAa represented an old locus originating from an ancestral DQA gene. Similar to the cow BoLA-DQA2, *Elda*-DQAb formed a separate branch in the intron-based trees, supporting that *Elda*-DQBb was an independent locus. Hence, we designated *Elda*-DQAa *Elda*-DQA1and *Elda*-DQAb *Elda*-DQA2, respectively. In the case of the DQBs, the intron 1-based trees clustered *Elda*-DQBb (100% confidence) with sheep *Ovar*-DQB2, while the intron 2-based trees grouped *Elda*-DQBb with cow BoLA-DQB1 and sheep *Ovar*-DQB1 into the same branch with bootstrap values of 100% (Figure 3K and 3L). For *Elda*-DQBa, it showed lower genetic similarity to *Elda*-DQBb than sheep *Ovar*-DQBs in all of the intron-based trees, suggesting impossible allelic relationship between two *Elda*-DQBs. Regardless of which clustering paradigm was used, these results revealed that *Elda*-DQBa and *Elda*-DQBb were two different loci, which we named *Elda*-DQB1 and *Elda*-DQB2.

For the DRBs, the intron 1-based NJ, MP, and Bayesian trees grouped DRBa and DRBd as the most closely related dyad, with bootstrap values higher than 95% (Figure 3G). However, the intron 2-based trees identified two closely related pairs, DRBa-DRBc and DRBc-DRBd, with bootstrap values higher than 95% in all three kinds of trees (Figure 3H). These findings are consistent with the fact that DRBa and DRBc are part of the H1 haplotype, while DRBb and DRBd are part of H2. Thus, the highly supported clustering of DRBa-DRBc and DRBb-DRBd in the intron 2-based trees can be seen as documenting two duplication events. In additional, the *Elda*-DRBs clustered confidently with the cow BoLA-DRB3 and sheep *Ovar*-DRB1 in all of the intron-based trees (Figures 3G and 3H), suggesting that the *Elda*-DRBs originated from an ancestral ruminant DRB gene corresponding to the current cow BoLA-DRB3 and sheep *Ovar*-DRB1. In view of pairing between DRBa and DRBd in the intron 1-based trees, we then further analyzed the phylogenetic relationships of these DRB sequences based on their 5′-UTR through exon 1. The NJ, MP, and Bayesian trees clustered DRBd together with cow BoLA-DRB3 and sheep *Ovar*-DRB (>60% bootstrap values), while the DRBa, DRBb and DRBc were grouped into the same branch (>60%; Figure 3F), suggesting that the DRBa and DRBd did not share an allelic relationship. Collectively, the phylogenetic trees and above-mentioned sequence characteristics all supported the notion that DRBa, DRBb, DRBc, and DRBd were all independent loci. Therefore, we termed these loci *Elda*-DRB1, *Elda*-DRB2, *Elda*-DRB3, and *Elda*-DRB4, respectively.

#### Gene composition of *Elda*-MHC class II multi-locus haplotypes

Combining this information with the above-described results from the four gene categories, we concluded that the two multi-locus MHC class II haplotypes corresponded to: DRA1*01 ∼ DRB1 ∼ DRB3 ∼ DQA1 ∼ DQB2 (H1), and DRA1*02 ∼ DRB2 ∼ DRB4 ∼ DQA2 ∼ DQB1 (H2). It is rather surprising that although the Père David's deer possessed nine MHC class II loci (one dimorphic DRA and a total of eight monomorphic DRB, DQA, and DQB genes), it showed only two polymorphic haplotypes of five genes. This suggests that the species had a historically strong immunological basis for survival, but now suffers from a weaker survival ability due to bottleneck-associated losses of diversity.

### Variability and selection of the *Elda*-MHC class II genes in the Beijing and Dafeng populations

Within the *Elda*-DRA locus, the two DRA alleles differed by only three nucleotides, and these were found at their cytoplasmic tails rather than in their antigen-presenting regions (Figure 2A). Furthermore, among the DRAs from different species, the observed amino acid variations in their antigen-presenting regions were generally found in non antigen-binding sites (Figure 2A), indicating that purifying selection has dominated the evolution of the DRA locus. In contrast, we identified 3–38 nucleotide acid (1–25 amino acid), 40 (20), and 31 (18) differences in the antigen-presenting exon 2 regions of the *Elda*-DRB, -DQA and -DQB loci, respectively. Most of these changes were located at or near the antigen-binding sites (Figures 2B-D), suggesting the presence of positive selection. The monomorphic nature of the *Elda*-DRBs, -DQAs and -DQBs meant that we were unable to estimate their allelic frequencies and non-synonymous/synonymous substitutions in studied populations. Instead, we evaluated the haplotype frequencies of H1 and H2, and found that they were 0.345 and 0.655, respectively, in the Beijing population, and 0.313 and 0.687, respectively, in the Dafeng population. The observed and expected heterozygosities were 0.487 and 0.454 (*P* = 0.542), respectively, for the Beijing deer, and 0.500 and 0.443 (*P* = 1), respectively, for the Dafeng deer; thus, both showed slightly higher than expected heterozygosities, but conformed to Hardy-Weinberg equilibrium. Moreover, when we subjected the haplotypic data to the Ewens-Watterson neutrality test, we obtained significances of 0.104 and 0.131 for the Beijing and Dafeng deer, respectively. This indicates that there was no evidence of balancing selection at the level of the multi-locus *Elda*-MHC haplotypes.

## Discussion

### Advantages of the novel protocol

One objective of this study was to develop universal procedures capable of isolating MHC class II genes from different mammals in a manner that could be easily repeated by other researchers. Rather than PCR-based locus isolation via homologous primers, during which loci may be missed due to primer mismatching, we used magnetic bead-based DNA hybridization of universal probes (Figure 1A). We began by designing universal primers for PCR-based probe synthesis (Figure 1A). Our ability to successfully generate biotin-labeled MHC class II gene probes from both the carnivorous giant panda and the herbivorous Père David's deer proved that our universal primers are versatile. Our novel protocol, which we call the HURRAH method, involves cDNA synthesis followed by the direct identification of functional MHC genes, thereby avoiding interference by pseudogenes. We utilized the SMART-PCR technique to generate high-yield full-length ds cDNAs that could support efficient hybridization (Figure 1A). We observed differences in the positive clone rates among the various probes and hybridizations, but if the positive clone rate for a particular probe is too low, the SMART-adaptor based PCR, magnetic bead-based isolation, and hybridization may be repeated until the required positive isolation rate is attained. To verify that all relevant sequences had been isolated, we used conventional reverse-transcribed control cDNA to reconstitute the SSCP-HD profiles of the mixed cDNA sequences obtained from the SMART-based MHC cDNA library (Figure 1A). For the first part of the protocol (HUR; Figure 1A), we focused on isolating the members of four MHC gene categories (DRA, DRB, DQA and DQB). This is because DP pseudogenes have been identified in a majority of mammals.

In the second part of the protocol (RAH), we used LR PCR to isolate genomic DNA spanning from exon 1 to exon 3 of the MHC genes, used it to obtain the intronic sequences flanking exon 2, and designed the appropriate cg-series primers (Figure 1B). We validated the ability of the cg-series primers to amplify all expressed MHC sequences, and then used these primers to scan the identified multi-locus haplotype homozygotes (i.e., the individuals that showed the simplest HD patterns for a given gene category). In cases where we obtained a very simple HD profile (such as one HD band), we used direct sequencing to examine whether the single band represented two or more closely related sequences that differed by only a few nucleotides. As an example, *Elda*-DRA1*01 and *Elda*-DRA1*02 differed by only a single nucleotide in the flanking region of exon 2, and were visualized as only one HD band in the DRA heterozygotes (Figure S3). Similarly, *Elda*-DRB2 and *Elda*-DRB4 differed by only three consecutive nucleotide changes, and appeared as a single mixed HD band (Figure S3). In these cases, however, the sequences could generally be distinguished based on their single-strand confirmations, as assessed using SSCP. Indeed, individuals homozygous for *Elda*-DRA1*01 and *Elda*-DRA1*02 had largely discrepant SSCP banding patterns (Figure S3), while those homozygous for *Elda*-DRB2 had a slightly elevated SSCP band relative to that representing *Elda*-DRB4 homozygotes (Figure S3). This is why we chose to use both SSCP and HD for mutational scanning. SSCP relies on the three-dimensional structure of ss DNA, which is highly sequence dependent and can reflect small sequence differences through gel mobility shifts [26]. The HDs, in contrast, are hybrid ds DNA molecules that are largely matched but differ by one or more mismatched base pairs. The mismatched bases induce bubbles in the HDs, reducing the mobility of HDs relative to homoduplexes [26]. Therefore, mixing the isolated sequences to reconstitute both the SSCP and HD profiles of each homozygous haplotype allowed us to ensure that we had isolated all of the relevant genes (Figure 1B).

Although the genomic sequences of the MHC genes are largely unknown for wild animals, various MHC cDNA sequences have been isolated from such animals, and a few studies have defined separate loci based on these cDNA sequences (Table 1). The main obstacles limiting our ability to identify loci from abundant cDNAs are the high degree of similarity among their non-antigen-presenting exons, and the lack of distinguishable intronic sequences. The polymorphisms seen in antigen-presenting exon 2 are driven by pathogens and therefore do not represent distinct loci. Although pedigree information has been used successfully to identify DRB haplotypes based on cDNA sequences from the red deer [27], few (if any) pedigree records are available for wild and semi-wild animals. In this study, we isolated all of the expressed full-length MHC cDNA sequences, and constructed NJ, MP, and Bayesian phylogenetic trees based on a large amounts of data, including the sequences of exon 3 through exon 5, the 5′-UTR through exon 1, intron 1, and intron 2. Our analysis of the sequences and phylogenetic relationships of the 10 putative *Elda*-MHC molecules revealed that the severely bottlenecked Père David's deer possessed nine MHC class II loci constituting two multi-locus haplotypes (DRA1*01 ∼ DRB1 ∼ DRB3 ∼ DQA1 ∼ DQB2 and DRA1*02 ∼ DRB2 ∼ DRB4 ∼ DQA2 ∼ DQB1). This is the first time that a complete multi-locus haplotype of MHC class II genes has been described for a wild animal without the laborious construction of a BAC library. Given its benefits over the previous methods, our novel technique could greatly facilitate future work in wild animal genetics.

### Development of the MHC class II genes in the Père David's deer

**Figure 4:**
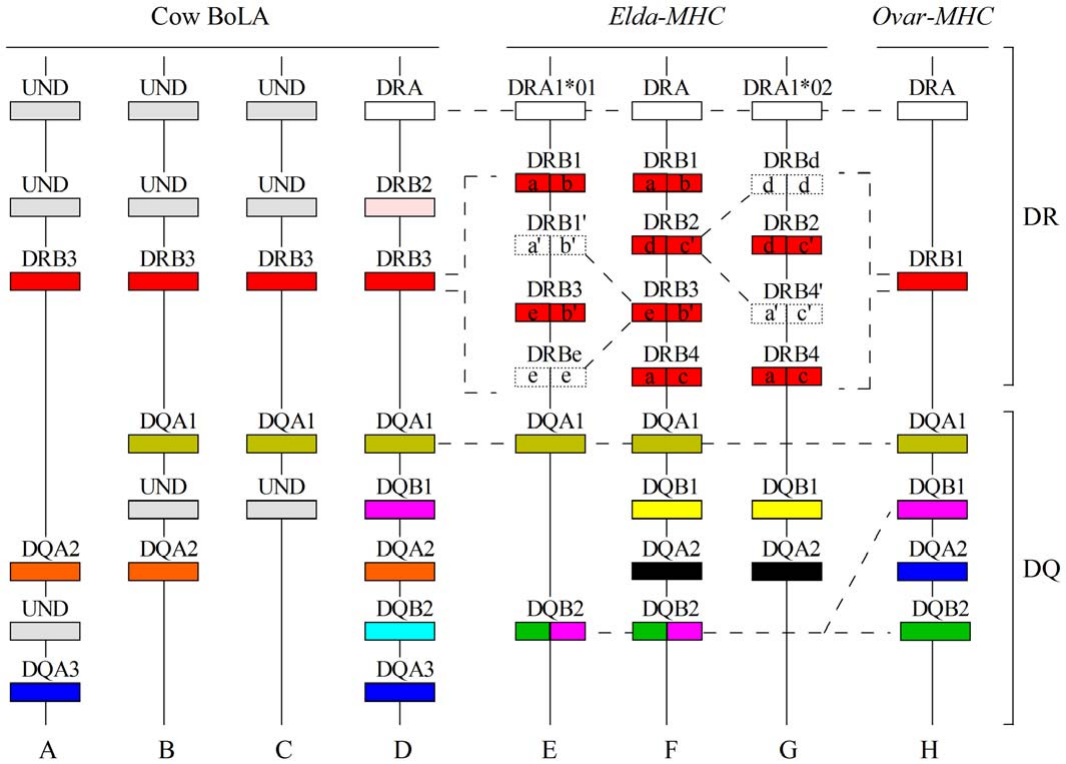
Schematic depiction of the evolution of the *Elda*-MHC class II genes. For comparison, the BoLA- and *Ovar*-MHC genes were included. The four BoLA class II multi-locus haplotypes shown (ABCD) are based on Ballingall et al. [28], Ellis and Ballingall [29], and the *Bos taurus* genome sequencing project (NCBI Btau 3.1). The *Ovar*-MHC class II multi-locus haplotype shown was revised according to Herrmann-Hoesing et al. [10]. Recombination events are shown by the dashed-line marked conversion of two adjacent rectangles, which represent intron 1 (left) and intron 2 (right). The colors show corresponding relationships among the MHC class II loci in the three ruminants. Dotted rectangles indicate historical existence of inferred genes. UND indicates “undetected.” We also show a powerful ancestral *Elda*-MHC class II haplotype (F) containing all of the identified loci.

In an effort to understand the MHC gene development of the target species, we constructed phylogenetic trees of *Elda*-MHC genes with reference to human cDNA sequences, and further included cDNA sequences from two ruminants: the cow and the sheep. In the NJ, MP and Bayesian trees generated based on the exon 3-exon 5 cDNA sequences, each *Elda*-MHC gene clustered separately (Figures 3A-D) and no relationships were observed with the known BoLA and *Ovar*-MHC loci. Notably, the trees constructed based on intron 1, intron 2 and the 5′-UTR through exon 1 differed from the exon 3-exon 5-based trees for the DRBs, DQAs, and DQBs, whereas the intron-based trees for the DRAs were consistent with those generated using the coding sequence (Figure 3). The conversions of (i.e. differences in) branches between the intron 1- and intron 2-based trees could be taken as reflecting recombination events that had occurred either within the Père David's deer (for *Elda*-DRBs), or among the three examined ruminant species (for DQB) (Figure 3). Based on these highly supported recombinatorial relationships, we created a schematic depicting the development of the *Elda*-MHC class II genes (Figure 4).

Among the *Elda*-DRBs, *Elda*-DRB1 and -DRB3 are linked within the H1 haplotype (Figure 4E), while *Elda*-DRB2 and -DRB4 are linked within H2 (Figure 4G). The highly supported pairings in the intron-based trees indicated that *Elda*-DRB1 ∼ DRB3 or *Elda*-DRB2 ∼ DRB4 arose from duplication events (Figures 4E-G). However, in the intron 1-based tree, *Elda*-DRB4 was not with its sister *Elda*-DRB2, but rather with *Elda*-DRB1 (Figures 3G), reflecting that the fragment upstream of *Elda*-DRB4 or *Elda*-DRB1 exon 2 had experienced a recombination event. Furthermore, the *Elda*-DRB2 and -DRB3 formed separate branches of themselves, suggesting that their original duplicated copies (*Elda*-DRB1′ and -DRB4′) underwent recombination events with different genes (which we designated *Elda*-DRBe and -DRBd) after duplication events (Figure 4E–G). The intron-based trees further showed that BoLA-DRB3 and *Ovar*-DRB1 were always paired together, and appeared at the base of the *Elda*-DRB cluster (Figures 3G and 3H), indicating that the four *Elda*-DRB genes had expanded from an ancestral gene corresponding to the current BoLA-DRB3 or *Ovar*-DRB1 (Figures 4E and 4G).

Regarding the DQA genes, *Elda*-DQA1 always clustered with BoLA-DQA1 and *Ovar*-DQA1 in the intron 1 and -2-based NJ, MP and Bayesian trees (Figures 3I and 3J), suggesting that *Elda*-DQA1, BoLA-DQA1, and *Ovar*-DQA1 had originated from the same ancestral DQA locus (Figures 4E–H). *Elda*-DQA2 existed as a separate branch in the intron 1- and intron 2-based trees (Figures 3I and 3J), suggesting that *Elda*-DQA2 arose from a distinct ancestral gene (Figure 4F and 4G). *Ovar*-DQA2 showed no relationship with the *Elda*-DQAs, but was paired with BoLA-DQA3 in both of the intron-based trees (>90% bootstrap values; Figures 3I and 3J), indicating that *Ovar*-DQA2 and BoLA-DQA3 arose from the same DQA ancestor (Figures 4D and 4H).

As for the DQB genes, BoLA-DQB1 and *Ovar*-DQB1 always clustered together (Figures 4K and 4L), suggesting that they shared a common DQB ancestor. *Elda*-DQB1 produced a separate branch in the intron 1- and intron 2-based trees (Figures 3K and 3L), suggesting that *Elda*-DQB1 developed from a distinct ancestral gene (Figure 4F and 4G). *Elda*-DQB2 clustered strongly with *Ovar*-DQB2 in the intron 1-based trees, but with BoLA-DQB1 and *Ovar*-DQB1 in the intron 2-based trees (Figures 3K and 3L), suggesting that *Elda*-DQB2 arose through recombinations among two ancestral DQB genes corresponding to BoLA-DQB1/*Ovar*-DQB1 and Ovar-DQB2 (Figures 4D–H). Collectively, these findings indicate that the various *Elda*-DRB and DQB loci underwent recombination events, further suggesting that the MHC region has historically been a recombination hotspot in the Père David's deer.

Haplotype polymorphism has been reported to exist in cow BoLA [28]–[29]; one haplotype has a single DQA1 locus (Figure 4C), one possesses DQA1 and DQA2 (Figure 4B), one has DQA2 and DQA3 (Figure 4A), and one has DQA1, DQA2, and DQA3 (NCBI Btau 3.1; 21229140∼21568700; Figure 4D). Similarly, Bowen et al. [30] inferred the existence of a seriously unequal number of DRB loci in California sea lions based on the number of cDNA sequences isolated from various individuals, which were found to express from three to seven DRB genes in peripheral blood. These examples show that although some haplotypes may comprise only a few loci, others can incorporate numerous loci from different haplotypes. Here, we note the following: (1) We inferred the historical existence of *Elda*-DRB1′, *Elda*-DRB4′, *Elda*-DRBd and *Elda*-DRBe (Figures 4E and 4G). (2) During our LR PCR experiments, we found evidence for the existence of an *Elda*-DQB3 pseudogene (data not shown). (3) We observed serious mismatching of MHC class II loci between the H1 and H2 haplotypes. Thus, we propose that the bottlenecked Père David's deer has lost the more powerful ancestral MHC class II haplotypes, which may have contained variants at all nine MHC class II loci (or even more).

### Variability of the MHC class II genes in the Père David's deer

We found that the sampled Père David's deer possessed 10 MHC class II sequences and had evolved a total of nine *Elda*-MHC loci, including *Elda*-DRA1 (two alleles resulting from a polymorphic cytoplasmic tail: *Elda*-DRA1*01 and *Elda*-DRA1*02), *Elda*-DRB1, *Elda*-DRB2, *Elda*-DRB3, *Elda*-DRB4, *Elda*-DQA1, *Elda*-DQA2, *Elda*-DQB1, and *Elda*-DQB2. Comparison of these results with those from the eight genome-sequenced mammals for which the full MHC data are available (Table 2) revealed that the Père David's deer had the highest known number of functional MHC class II loci, suggesting that this deer should have a strong species survival ability. However, all of the *Elda*-MHC class II loci were monomorphic at their antigen-binding regions, suggesting that this deer species currently maintains a relatively weak antigen-presenting ability. This apparent contradiction may provide new insight into the unique evolutionary history of this deer. Our results from the novel HURRAH protocol indicated that this deer was polymorphic at the level of its multi-locus haplotypes, with the dimorphic *Elda*-DRA and eight monomorphic DRB, DQA and DQB loci assembling into two functional MHC class II multi-locus haplotypes: DRA1*01 ∼ DRB1 ∼ DRB3 ∼ DQA1 ∼ DQB2 (H1) and DRA1*02 ∼ DRB2 ∼ DRB4 ∼ DQA2 ∼ DQB1 (H2). Furthermore, the presence of serious mismatching of MHC class II loci between these two haplotypes suggested that some powerful ancestral MHC class II haplotypes containing more loci may have once existed in the Père David's deer.

**Table 2:** Comparison of the number of MHC class II loci expressed among different mammals.

Species	DRA	DRB	DQA	DQB	Number of loci	Reference
Père David's deer	1	4	2	2	9	This study
Human	1	2	2	1	6	[5]
Mouse	1	1	1	1	4	[4]
Cow	1	2	3	2	8	NCBI Btau 3.1
Sheep	1	1	2	2	6	[10]
Pig	1	1	1	1	4	[9]
Cat	3	3	0	0	6	[6]
Dog	1	1	1	1	4	[8]
Giant panda	1	1	2	2	6	[34]; this study

Previous studies of highly variable microsatellite repeats revealed limited genetic variation (two to three alleles) in the Père David's deer [31]–[32], suggesting the presence of serious bottleneck effects. Similarly, we herein identified only two MHC class II multi-locus haplotypes in this species, suggesting that some MHC haplotypes may have been lost through the known bottlenecks, perhaps at times when permissive conditions might have caused the MHC loci to act like neutral loci. In addition, the maintenance of two MHC class II multi-locus haplotypes in this deer could be partially attributed to herd resistance and conservation efforts aimed at sustaining the founder population. Regardless, the HURRAH results suggest that the Père David's deer may have lost some powerful ancestral MHC class II haplotypes of nine or more loci, and have fixed two relatively weak haplotypes containing five genes. As a result, the current Père David's deer should be considered to be at risk for an increased susceptibility to infectious pathogens. We therefore recommend that managers should seek to design and implement scientific reproduction strategies aimed at increasing the number of haplotype heterozygotes (H1/H2) in the deer descendants, in order to enhance the survival ability of this species. Furthermore, when managers seek to build new populations, they should fully evaluate the genetic structure of the founders and ensure that haplotype heterozygotes are selected.

## Materials and Methods

### Ethics statement

The blood and skin samples of living animals in this study were provided to us by the institutional staff during routine examinations (blood) and marking of newborn calves (ear notches) so that no ethics statement is required.

### Sampling

During 2005–2008, samples were collected from a total of 220 Père David's deer; of them, 153 specimens (25 skin and 128 blood samples, all taken from a previous study of Zhang et al. [33]) were obtained from the semi-wild population in Beijing Nanhaizi Milu Park, while the remaining 67 individuals (four blood and 63 fecal samples) were drawn from the Dafeng Milu National Nature Reserve. The Beijing Nanhaizi Milu Park and Dafeng Milu National Nature Reserve populations should be considered the founder populations for all of the Père David's deer herds in China, and thus should represent the genetic variations in the offspring populations.

### Isolation of cDNA sequences

#### Extraction of mRNA and synthesis of ds cDNA

Total RNA was extracted from fresh blood, liver and brain samples using the TRIzol Total RNA Isolation Kit (Invitrogen), and mRNA was purified employing an Oligotex mRNA Spin-Column Kit (Qiagen). Full-length cDNA was generated using a SMART™ PCR cDNA Synthesis Kit (Clontech) according to the manufacturer's instructions (http://www.clontech.com). Partial mRNA was reverse-transcribed to generate PCR controls for the SMART cDNA using the MMLV First-strand cDNA Synthesis Kit (Sangon). PCR amplification was carried out using an Applied Biosystems 2720 thermal cycler (Applied Biosystems).

#### Design of universal primers

Although the MHC class II region may contain DR, DQ, and DP genes, only humans show functional DP subregions; the DPA and DPB loci of other mammals have evolved into pseudogenes [34]. Therefore, only the DR and DQ genes were included in this study. To design universal primers for PCR-based probe synthesis, we used the full-length cDNA sequences of HLA-DRA, -DRB, -DQA, and -DQB as references, and downloaded the 100 sequences that showed the highest homologies to each HLA gene, as revealed by NCBI Blast search results. We then aligned each group of 100 sequences using the ClustalW algorithm in the Mega 3.1 software package [35]. We omitted any overly short sequences, and then searched among the remaining sequences for monomorphic or oligomorphic sites. We identified the most conserved regions containing multiple successive monomorphic/oligomorphic sites, and used these sequences to design pairs of universal primers (u-series) for DRA, DRB, DQA, and DQB (Table S1), using the PrimerSelect software package (DNAstar). All of the u-series primers yielded products when used for PCR amplification of DNA from the giant panda and the Père David's deer.

#### Preparation of biotinylated probes

The probes were biotinylated in a 25 µL PCR reaction system containing 1 µL 10 ng/µL template DNA, 1 U of Ex Taq DNA polymerase (TaKaRa), 2.5 µL 10×PCR buffer, 2 µL 25 mM MgCl_2_, 1 µL of 10 µM u-series primers, 1 µL 5 mM dATP, 1 µL 5 mM dCTP, 1 µL 5 mM dGTP, 0.75 µL 5 mM dTTP, and 1.25 µL 1 mM Bio-11-dUTP (Fermentas [MBI]). To measure the efficiency of biotin labeling, we established a 25 µL PCR control tube containing the above reagents with the exception of the Bio-11-dUTP and the addition of 2 µL dNTPs (10 mM each). The reaction conditions consisted of a 5 min denaturation step at 95°C, followed by 30 cycles of 30 s at 95°C, 40 s at the appropriate optimized annealing temperature (Table S1), 30 s at 72°C, and a final extension at 72°C for 10 min. The resulting PCR products were visualized by agarose gel electrophoresis.

#### Magnetic bead-based cDNA hybridization

Generally, the full-length cDNAs from MHC class II exons are larger than 750 base pairs (bp) [4]–[10]. Thus, 750–2,000 bp-sized fragments were isolated from ds cDNA generated by the SMART™ PCR cDNA Synthesis Kit (Clontech), and these fragments were hybridized with the biotinylated probes. Hybridization was carried out in a 100 µL volume containing 10 µL of 10 ng/µL biotinylated probe, 10 µL of 80 ng/µL ds cDNA, and 50 µL of 12×SSC (saline sodium citrate; 150 mM NaCl, 15 mM sodium citrate, 0.1% [w/v] SDS; pH 7.0). The ds cDNA mixture was denatured at 95°C for 10 min, and then incubated for 5 h at 60°C (for DRA and DQB) or 65°C (for DRB and DQA). The hybridized MHC cDNA fragments were captured using streptavidin-coated magnetic beads (Promega) by virtue of the probe-linked biotin. To convert the eluted ss DNA into ds DNA and increase the amount of material available for analysis, the enriched fragments were amplified using the SMART adaptors as primers, according to the manufacturer's instructions (Clontech). The resulting PCR products were cloned into the pMD-18T vector (TaKaRa) for construction of the MHC-enriched cDNA library. Twenty randomly selected positive clones from the first two independent hybridizations (10 per hybridization) were bidirectionally sequenced on an ABI 3730 automated sequencer (Applied Biosystems).

#### Design of cc-series primers

The cDNA sequences were assembled using the SeqMan software package (DNAstar), which yielded an output consensus for each representative sequence. We then aligned these sequences with the publicly available cDNA sequences used in the design of u-series primers (Table S1), and identified conserved flanking regions of the hypervariable exon 2 that could be used to design the cc-series primers. The DRAs of the Père David's deer were found to be identical at antigen-presenting exon 2; however, they differed at exon 4, so the cc-series primers of *Elda*-DRA were localized to exon 3 and the 3′-UTR. All of the other cc-series primers localized to exons 1 and 3 for the Père David's deer (Table S2). The cc-series PCR products were then used to reconstitute the various SSCP and HD profiles. Once the SSCP-HD banding patterns were reconstituted successfully, the cDNA sequences were subjected to additional primer design for isolation of the corresponding genomic sequences spanning exon 1 to exon 3.

#### Profile reconstitution of SSCP-HD banding patterns at the levels of cDNA

SSCP and HD are two mutation-scanning techniques that may be run on the same polyacrylamide gel [26], and therefore do not require separate experiments. The SSCP-HD procedures were performed routinely using a DCode Universal Mutation Detection System (Bio-Rad) according to the manufacturer's instructions. All cc- and cg-series PCR products (Table S2) were first purified using a PCR Purification Kit (V-gene), and then mixed with SSCP loading buffer. The sample mixtures were heated at 95°C for 5 min and subsequently chilled on ice. The resulting denatured PCR products were run on a nondenaturing polyacrylamide gel, and gel silver staining was performed as described by Beidler et al. [36]. When the desired band intensity was reached, the gel was fixed in pre-chilled acetic acid and dried for photography.

To reconstitute the cDNA-derived SSCP and HD banding patterns, we first used the cc-series PCR primers to amplify the SMART PCR products and conventional cDNAs, and compared their SSCP-HD banding patterns. When identical profiles were obtained, we concluded that the SMART cDNA effectively represented the original cDNA. We then used the cc-series primers to amplify clones representing the different MHC sequences, and mixed them to achieve compound SSCP-HD profiles, which we then compared with those obtained from regular cDNA. If the conventional cDNA presented more HD bands than the mixture, the newer HD bands were excised and subjected to cloning. Three to eight independent clones were sequenced for the determination of each novel sequence. After that, profile reconstitution was repeated with the newer sequences, until the SSCP-HD profiles were identical between the mixed and conventional samples. Finally, the standard SSCP-HD banding patterns of the new cc-series sequences were utilized to find the corresponding full-length SMART cDNA clones and obtain their full-length cDNA sequences.

### Isolation of genomic sequences

#### Extraction of genomic DNA

Genomic DNA was isolated from blood and skin samples by standard methods [37]. Fecal DNA was extracted as described by Wan et al. [38], with minor modifications. Specifically, fecal pellets were rehydrated for 1–2 hr in TNE buffer (10 mM Tris-HCl, 100 mM NaCl, 10 mM EDTA; pH 7.8). The outer mucosal layers of the wet pellets were carefully removed and placed in 50-mL tubes with 50-mL TNE buffer. Samples were incubated overnight with shaking, and then DNA extraction was performed according to the protocol described by Wan et al. [38].

#### Design of es-series primers

Matched 3′-end bases and unique 3′ sequences are prerequisites for primer design, because unmatched end bases and 3′ sequences affect the progression of Taq polymerase and primer binding, respectively, and can lead to amplification failure. These two primer features have been exploited to allow the amplification of only the desired nucleotide sequences and not other (unwanted) very similar DNAs [39]–[41]. We used this paradigm when designing es-series primers, with the aim of isolating the genomic sequences corresponding to each cDNA sequence (Table S3), which could then be used to distinguish loci from alleles. The MHC cDNA sequences of the Père David's deer were aligned using Mega 3.1 [35], and the polymorphic sites were identified. Based on the unique sequence variations within each MHC sequence, we designed primers to specifically amplify genomic sequences spanning exon 1, intron 1, exon 2, intron 2, and exon 3, using LR PCR and exon-localized primers (Table S3).

#### LR PCR and cg-series DNA primers

We performed LR PCR with LA Taq (TaKaRa) using a two-step cycling program that consisted of a 3 min denaturation step at 95°C, followed by 30 cycles of 30 s at 95°C, 2–8 min (for 2–8 kilobases) annealing and extension at 68°C (Table S3), and a final extension at 72°C for 10 min. A 50 µL PCR reaction mixture was prepared as suggested by the manufacturer. The LR PCR products were cloned using the Topo TA Cloning Kit (Invitrogen) according to the manufacturer's protocol. Each fragment was amplified in three separate PCR batches, and three independent clones per PCR reaction were chosen for sequencing. The sequences were assembled into a final consensus sequences using the SeqMan software (DNAStar). The intronic sequences from the Père David's deer MHC class II genes were aligned and used to design the cg-series primers (Table S2), which were then used to scan the simplest HD banding patterns. The most complex HD profile was sometimes also scanned for comparison.

#### Profile reconstitution of SSCP-HD banding patterns at the levels of genomic DNA

To recover the SSCP-HD banding patterns of genomic DNA from the Père David's deer, we used the cg-series primers to scan the most homozygous individuals, subjected the relevant PCR products to cloning and sequencing, and used the clones to reconstitute the SSCP-HD profiles as described above. Only successful reconstitution of SSCP-HD profiles can ensure isolation of all of the normal MHC genomic sequences. Furthermore, based on successful reconstitution of SSCP-HD banding patters of homozygous haplotypes, we could enumerate the functional MHC class II genes. All cg-series PCR products for SSCP-HD analysis were amplified using conventional PCR conditions and optimized annealing temperatures (Tables S2).

### Bioinformatic analysis and construction of MHC class II multi-locus haplotypes

The full-length cDNA sequences were aligned first using the ClustalW algorithm in Mega 3.1 [35]. The sequences were then trimmed at the start and stop codons, the amino acid sequences were deduced, and variations across the coding sequences were analyzed. The putative antigen-binding sites for each of the MHC class II genes were determined based on their orthologs in human HLA [42]. For construction of the phylogenetic trees, the intronic sequences were first run through the Repeatmasker program (http://www.repeatmasker.org), which masked evolutionarily non-neutral repeats. The exon 3-exon 5, 5′-UTR-exon 1, and repeat-masked intronic sequences were then separately aligned using the ClustalW algorithm in Mega 3.1 [35] and subjected to construction of NJ, MP and Bayesian trees using the Mega 3.1 [35], PAUP4.0b [43] and MRBAYES 3.1.2 [44] programs, respectively.

The MP trees were produced using heuristic searches with tree-bisection-reconnection (TBR) branch swapping, and node support was tested by bootstrapping of 10,000 replicates. As for the NJ and Bayesian trees,we deduced the appropriate models of evolution using FindModel web server (http://www.hiv.lanl.gov/content/sequence/findmodel/findmodel.html) and then constructed them in Mega and MRBAYES. Bootstrap values of the NJ trees were calculated using 10,000 replicates. The Bayesian phylogenetic analyses were performed by running Markov Chain Monte Carlo (MCMC) simulations for 10,000 generations, with four simultaneous chains, a sample frequency of 10, and a burn-in of 250 runs. The PyPop 0.7.0 software package [25], which was originally developed for analyzing haplotypes from the multi-locus genotype data of the highly polymorphic HLA locus [45], was employed to evaluate multi-locus *Elda*-MHC class II haplotypes, estimate haplotypic frequencies, and test for deviation from Hardy-Weinberg equilibrium using the Markov chain permutation test of 10,000 steps. The Ewens-Watterson homozygosity test was implemented in PyPop, and Slatkin's exact test [46] was used to obtain the probability of homozygosity under neutrality (10,000 iterations).

## Supporting Information

Data S1 Verification of the novel protocol using the giant panda.
(0.57 MB DOCX)

Figure S1 Conventional amplification and biotinylated probe preparation using u-series universal primers. A) PCR products from the giant panda. B) Comparison between normal PCR products and biotinylated probes (marked as DRAp, DRBp, DQAp, and DQBp). C) PCR products from the Père David's deer.
(0.15 MB TIF)

Figure S2 Profile reconstitutes of cDNA-derived SSCP-HD banding patterns based on cc-series PCR products from the Père David's deer, and their use to ensure the completeness of cDNA isolation. The numbers show the electrophoretic lanes, and the letters represent the cDNA sequences isolated. Abbreviations: C, control from conventional cDNA; M, a mix of the products shown in the lanes between C and M.
(0.41 MB TIF)

Figure S3 The SSCP-HD banding patterns for the cg-series PCR products of the Père David's deer. The same individuals were investigated for the DRA, DRB, DQA, and DQB genes. The banding patterns designated with letters a, b, c, d correspond to the cDNA sequences isolated. The symbols ▴, ▾, and ▴+▾ indicate the homozygous H1, homozygous H2 and heterozygous H1/H2 haplotypes, respectively. Here, we also show two mixed samples (lane 10 is a mixture of lanes 6 and 9, and lane 11 is a mixture of lanes 7 and 8) that reconstitute the H1/H2 heterozygote.
(1.53 MB TIF)

Figure S4 Genomic DNA-derived SSCP-HD banding patterns reconstituted using cg-series PCR products amplified from the Père David's deer. The homozygous DQA and DQB haplotypes genes each only represented one locus, so we chose to reconstitute the two homozygotes and one heterozygote of the Elda-DRB haplotype. Ear tag numbers 34, 4 and 1 are deer that showed the H1, H2 and H1/H2 DRB haplotypes, respectively. The information beside each *Elda*-MHC gene indicates the initial cDNA sequences (a, b, c and d) and the subsequently identified loci (1, 2, 3 and 4).
(0.50 MB TIF)

Table S1 Universal (u-series) primers used for probe preparation, which successfully amplified DNA from the carnivorous giant panda and the herbivorous Père David's deer.
(0.04 MB DOC)

Table S2 Common cDNA (cc-series) and genomic DNA (cg-series) primers for the Père David's deer; these were used to confirm that all relevant sequences had been isolated, as assessed by reconstitution of SSCP-HD profiles.
(0.04 MB DOC)

Table S3 Exon-specific (es-series) primers for the Père David's deer were used to isolate the genomic introns of each isolated cDNA sequence. All primer pairs were subjected to LR PCR, using 68°C as the annealing and extension temperature.
(0.05 MB DOC)

## References

[1] Klein J (1986). Natural history of the major histocompatibility complex..

[2] Marsh SGE, Parham P, Barber LD (2000). The HLA factsbook..

[3] König R, Shen X, Germain RN (1995). Involvement of both major histocompatibility complex class II α and β chains in CD4 function indicates a role for ordered oligomerization in T cell activation.. J Exp Med.

[4] Rowen L, Qin S, Loretz C, Mix L, Lasky S (1998). Sequence of the mouse major histocompatibility class II region..

[5] The MHC Sequencing Consortium (1999). Complete sequence and gene map of a human major histocompatibility complex.. Nature.

[6] Yuhki N, Beck T, Stephens RM, Nishigaki Y, Newmann K (2003). Comparative genome organization of human, murine and feline MHC class II region.. Genome Res.

[7] Childers CP, Newkirk HL, Honeycutt DA, Ramlachan N, Muzney DM (2005). Comparative analysis of the bovine MHC class IIb sequence identifies inversion breakpoints and three unexpected genes.. Anim Genet.

[8] Debenham SL, Hart EA, Ashurst JL, Howe KL, Quail MA (2005). Genomic sequence of the class II region of the canine MHC: comparison with the MHC of other mammalian species.. Genomics.

[9] Ando A, Chardon P (2006). Gene organization and polymorphism of the swine major histocompatibility complex.. Anim Sci J.

[10] Herrmann-Hoesing L, White S, Kappmeyer L, Herndon D, Knowles D (2008). Genomic analysis of *Ovis aries* (Ovar) MHC class IIa loci.. Immunogenetics.

[11] Jäger I, Eizaguirre C, Griffiths SW, Kalbe M, Krobbach CK (2007). Individual MHC class I and MHC class IIB diversities are associated with male and female reproductive traits in the three-spined stickleback.. J Evol Biol.

[12] Croisetière S, Tarte PD, Bernatchez L, Belhumeur P (2008). Identification of MHC class IIbeta resistance/susceptibility alleles to Aeromonas salmonicida in brook charr (*Salvelinus fontinalis*).. Mol Immunol.

[13] Loiseau C, Zoorob R, Garnier S, Birard J, Federici P (2008). Antagonistic effects of a Mhc class I allele on malaria-infected house sparrows.. Ecol Lett.

[14] Bernatchez L, Landry C (2003). MHC studies in nonmodel vertebrates: what have we learned about natural selection in 15 years?. J Mol Evol.

[15] Bonneaud C, Chastel O, Federici P, Westerdahl H, Sorci G (2006). Complex Mhc-based mate choice in a wild passerine.. Proc Biol Sci.

[16] Schwensow N, Eberle M, Sommer S (2008). Compatibility counts: MHC-associated mate choice in a wild promiscuous primate.. Proc Biol Sci USA.

[17] Mikko S, Røed K, Schmutz S, Andersson L (1999). Monomorphism and polymorphism at Mhc DRB loci in domestic and wild ruminants.. Immunol Rev.

[18] Gutierrez-Espeleta GA, Hedrick PW, Kalinowski ST, Garrigan D, Boyce WM (2001). Is the decline of desert bighorn sheep from infectious disease the result of low MHC variation?. Heredity.

[19] Babik W, Durka W, Radwan J (2005). Sequence diversity of the MHC DRB gene in the Eurasian beaver (*Castor fiber*).. Mol Ecol.

[20] Meyer-Lucht Y, Otten C, Püttker T, Sommer S (2008). Selection, diversity and evolutionary patterns of the MHC class II DAB in free-ranging Neotropical marsupials.. BMC Genet.

[21] Ohtaishi N, Gao Y (1990). A review of the distribution of all species of deer (Tragulidae, Moschidae and Cervidae) in China.. Mamm Rev.

[22] Ding YH (2004). Chinese milu research, Jinlin Publishing House for the Science and Technology, China.

[23] Jones ML, Manton VJA, Beck BB, Wemmer C (1983). History in captivity.. The biology and management of an extinct species: Père David's deer.

[24] Jiang Z, Feng Z, Yu C, Zhang L, Xia J (2000). Reintroduction and recovery of Pere Davidps deer in China.. Wildl Soc Bull.

[25] Lancaster AK, Single RM, Solberg OD, Nelson MP, Thomson G (2007). PyPop update - a software pipeline for large-scale multilocus population genomics.. Tissue Antigens.

[26] Wallace AJ, Theophilus BDM, Rapley R (2002). SSCP/Heteroduplex analysis..

[27] Swarbrick PA, Schwaiger FW, Epplen JT, Buchan GS, Griffin JF (1995). Cloning and sequencing of expressed DRB genes of the red deer (*Cervus elaphus*) Mhc.. Immunogenetics.

[28] Ballingall KT, Luyai A, McKeever DJ (1997). Analysis of genetic diversity at the DQA loci in African cattle: evidence for a BoLA-DQA3 locus.. Immunogenetics.

[29] Ellis SA, Ballingall KT (1999). Cattle MHC: evolution in action?. Immunol Rev.

[30] Bowen L, Aldridge BM, Gulland F, Van Bonn W, DeLong R (2004). Class II multiformity generated by variable MHC- DRB region configurations in the California sea lion (*Zalophus californianus*).. Immunogenetics.

[31] Jiao Y, Ge YF, Fang SG (2008). Eight novel microsatellite markers from the Pere David's deer (*Elaphurus davidianus*).. Conserv Genet.

[32] Wu HL, Ni XW, Zhang LY, Xia JS, Zhong ZY (2008). Eighteen novel polymorphic microsatellite loci developed from the Pere David's deer (*Elaphurus davidianus*).. Conserv Genet.

[33] Zhang LY, Wu HL, Zhong ZY, Zhang SM (2010). Microsatellite polymorphisms and population genetic structure of *Elaphurus davidianus* in Beijing Milu Park. Sichuan.. J of Zool.

[34] Wan QH, Zeng CJ, Ni XW, Pan HJ, Fang SG (2009). Giant panda genomic data provide insight into the birth-and-death process of mammalian major histocompatibility complex class II genes.. PLoS ONE.

[35] Kumar S, Dudley J, Nei M, Tamura K (2008). MEGA: A biologist-centric software for evolutionary analysis of DNA and protein sequences.. Brief Bioinform.

[36] Beidler JL, Hilliard PR, Rill RL (1982). Ultrasensitive staining of nucleic acids with silver.. Anal Biochem.

[37] Sambrook J, Russell DW (2001). Molecular cloning: a laboratory manual, 3rd edn..

[38] Wan QH, Zhu L, Wu H, Fang SG (2006). Major histocompatibility complex class II variation in the giant panda (*Ailuropoda melanoleuca*).. Mol Ecol.

[39] Wan QH, Fang SG (2003). Application of species-specific polymerase chain reaction in the forensic identification of tiger species.. Forensic Sci Int.

[40] Wan QH, Qian KX, Fang SG (2003). A simple DNA extraction and rapid specific identification technique for single cells and early embryos of two breeds of *Bos taurus*.. Anim Reprod Sci.

[41] Fernandes CA, Ginja C, Pereira I, Tenreiro R, Bruford MW (2008). Species-specific mitochondrial DNA markers for identification of non-invasive samples from sympatric carnivores in the Iberian Peninsula.. Conserv Genet.

[42] Reche PA, Reinherz EL (2003). Sequence variability analysis of human class I and Class II MHC molecules: functional and structural correlates of amino acid polymorphisms.. J Mol Biol.

[43] Swofford DL (1998). PAUP*. Phylogenetic Analysis Using Parsimony (*and Other Methods). Version 4..

[44] Ronquist F, Huelsenbeck JP (2003). MrBayes 3: Bayesian phylogenetic inference under mixed models.. Bioinformatics.

[45] Lancaster A, Nelson M, Meyer D, Single R, Thomson G (2003). PyPop: a software framework for population genomics: analyzing large-scale multi-locus genotype data.. In: Pacific Symposium on Biocomputing.

[46] Slatkin M (1994). An exact test for neutrality based on the Ewens sampling distribution.. Genetical Res.

[47] Swarbrick PA, Crawford AM (1997). The red deer (*Cervus elaphus*) contains two expressed major histocompatibility complex class II DQB genes.. Anim Genet.

[48] Yang W-C, Chou L-S, Hu J-M (2007). Molecular characterization of major histocompatibility complex class II DQB and DRB genes in bottleneck dolphins (*Tursiops truncatus* and *T. aduncus*) from the Western Pacific.. Zool Studies.

[49] Heimeier D, Baker CS, Russell K, Duignan PJ, Hutt A (2009). Confirmed expression of MHC class I and class II genes in the New Zealand endemic Hector's dolphin (*Cephalorhynchus hectori*).. Mar Mamm Sci.

[50] Heinzelmann LS, Tavares M, Ott PH, Moreno IMB, Chies JAB (2009). MHC class II expression in skin biopsies from the franciscana dolphin Pontoporia blainvillei and the southern right whale *Eubalaena australis*.. J Mar Biolog Assoc UK.

[51] Niranjan SK, Deb SM, Sharma A, Mitra A (2009). Isolation of two cDNAs encoding MHC-DQA1 and -DQA2 from the water buffalo, *Bubalus bubalis*.. Vet Immunol Immunopathol.

[52] Takada T, Kikkawa Y, Yonekawa H, Amano T (1998). Analysis of goat MHC class II DRA and DRB genes: identification of the expressed gene and new DRB alleles.. Immunogenetics.

[53] Schaschl H, Goodman SJ, Suchentrunk F (2004). Sequence analysis of the MHC class II DRB alleles in Alpine chamois (*Rupicapra r. rupicapra*).. Dev Comp Immunol.

[54] Bowen L, Aldridge BM, Gulland F, Woo J, Van Bonn W (2004). Molecular characterization of expressed DQA and DQB genes in the California sea lion (*Zalophus californianus*).. Immunogenetics.

[55] Vage DI, Olsaker I, Ronningen K, Lie O (1994). Partial sequence of an expressed major histocompatibility complex gene (DQA) from artic fox (*Alopex lagopus*).. Anim Biotechnol.

