# Supplementary material for: A Novel HURRAH Protocol Reveals High Numbers of Monomorphic MHC Class II Loci and Two Asymmetric Multi-Locus Haplotypes in the Père David's Deer
Source: PLoS One. 2011 Jan 18;6(1):e14518. doi: 10.1371/journal.pone.0014518 (PMC3022581; doi:10.1371/journal.pone.0014518)

**Supplementary data S1**

**Verification of the novel protocol using the giant panda**

**Supplementary Introduction**

The giant panda is a flagship animal of the international conservation movement, and has thus received a great deal of attention from wildlife managers and conservation biologists. At present, the giant panda is the only wild animal for which complete MHC-genome data are available; indeed, our group performed the necessary library construction, physical map development, and genome sequencing [S1-S2]. In the present study, we used this information from the giant panda to validate the efficiency of a novel protocol for isolating functional MHC class II genes.

**Supplementary Results**

Isolation of the *Aime*-MHC class II cDNA sequences

The HURRAH protocol revealed seven, seven and eight MHC class II sequences in samples taken from three panda individuals, named Chenggong, Jiaozizhizi, and Eryatouzhizi (Figures S1A-C and Table S1). The mRNA samples derived from the peripheral blood of Chenggong and the liver of Jiaozizhizi yielded similar results, showing one DRA, two DRB, two DQA, and two DQB sequences. In contrast, mRNA derived from the brain of Eryatouzhizi yielded an additional DQA sequence (Figure S1 and Table S1). In total, the three individuals yielded thirteen different cDNA sequences: one DRA sequence (termed DRAa), four DRB sequences (DRBa, DRBb, DRBc, and DRBd), four DQA sequences (DQAa, DQAb, DQAc, and DQAd), and four DQB sequences (DQBa, DQBb, DQBc and DQBd).

The SSCP and HD banding patterns of each gene type were successfully reconstituted for each individual (Figure S1), indicating that we had successfully isolated all of the expressed MHC sequences. Interestingly, the results obtained from the HURRAH method (Table S1) suggested that there was expression of one DRA, one DRB, two DQA, and two DQB genes in the giant panda. This was inconsistent with a previous report indicating the presence of two functional DRB loci in this species [S3]. When we aligned our identified sequences with the previously reported MHC sequences, we found the following: (1) For gene categories DRB, DQA and DQB, with the exception of antigen-presenting exon 2, the trans-membrane and cytoplasmic regions were identical (at both the nucleotide and amino acid levels) for all four DRBs, for all four DQBs, and for DQAa, DQAb and DQAc, but not DQAd (Figure S2). (2) The sequences we had designated DRAa, DRBa, DRBc, DQAa, DQAb, DQAc, DQAd, DQBa, DQBb, DQBc, and DQBd corresponded to the previously reported alleles DRA1*01, DRB3*01, DRB3*02, DQA1*02, DQA1*03, DQA1*05, DQA2*01, DQB1*01, DQB1*04, DQB1*02, and DQB2*01 [S3], respectively (Table S1). (3) The DRBb and DRBd sequences showed some nucleotide similarities to the previously reported DRB1*02 and DRB1*01 sequences but were not identical, indicating that DRBb and DRBd represented two new DRB sequences. Therefore, we obtained several unexpected results: (1) We did not detect the “functional” DRB1 that had been previously identified as expressed based on analysis of exon 2 sequences [S4]. (2) We did not detect the three DRB sequences that had been identified as being expressed in a study using exon 2-based 3’ RACE (rapid amplification of cDNA ends) [S3]. (3) The brain of Eryatouzhizi showed mRNA expression of the functional DQA2 predicted by partial genomic sequencing of the MHC genes [S2], but the blood from Chonggong and the liver of Jiaozizhizi did not. (4) It is presently unclear which DRB locus is the parental gene for DRBb and DRBd.

Characteristics of amino acid sequences deduced from the *Aime*-MHC class II sequences

Our analysis of the deduced amino acid sequences of the thirteen giant panda cDNA sequences obtained herein showed that all of the genes possessed normal initiation and termination signals except for DQA2*01 (Figure S2), which had a shorter signal peptide and a cytoplasmic tail, and therefore had the characteristics of a non-classical MHC gene [S6]. This finding, combined with our observation that DQA2 was present in the brain but not in blood or liver, may indicate that the functional DQA2 has evolved into a tissue-specific, non-classical MHC class II gene (Table S1).

Comparison between cDNA and BAC sequences

SSCP and sequence analysis of variable exon 2, along with comparison of cDNA and BAC sequences (Figure S3A), allowed us to reach the following conclusions: (1) The functional DRB gene predicted by partial genome sequencing of the MHC region was, indeed, expressed; it corresponded to the *Aime*-DRB3 reported by Chen et al. [S3]. (2) The newly identified DRBb and DRBd sequences are alleles of DRB3, which were not identified in the study of Chen et al. [S3], and were thus re-named DRB3*07 and DRB3*08 (Figure S3A and Table S1). (3) DRB1 was a pseudogene located outside of the previously sequenced contig (Figure S3A). (4) The rough classification of functional genes obtained from our HURRAH results was consistent with from the BAC contig, with one DRA, one DRB, two DQA, and two DQB genes identified (Figure S3A and Table S1). (5) Multiple copies of only the DQB gene were present in the same BAC (i.e., 237E1 and 1113F7; Figure S3A). (6) Using the HURRAH protocol, the classical MHC class II genes could be reliably identified from peripheral blood mRNA, which is relatively easy to sample.

Construction of *Aime*-MHC class II multi-locus haplotypes

Considering that the only gene found as a double copy in a single BAC was DQB (Figure S3A), we choose to reconstitute the SSCP-HD profiles obtained using the cg-series primers for DQB (Table S2). The resulting amplicons from 40 giant pandas yielded four versions of the simplest (three-band) HD banding pattern; three were new patterns, while one corresponded to that obtained from BACs 237E1 and 1113F7 (Figure S3B). The reconstituted profiles revealed that the simplest pattern from the BACs was composed of DQB1*04 and DQB2*01, while other three most homozygous individuals represented DQB1*01+DQB2*01 (Figure S3B), DQB1*03+DQB2*01 (Figure S3C), and DQB1*02+DQB2*01 (not shown) haplotype homozygotes. Thus, the simplest HD banding patterns appeared to be very useful for identifying homozygous haplotypes and estimating the number of MHC class II genes. In addition, they could be used to construct multi-locus haplotypes of the DQB gene category from the homozygous individuals, such as DQB1*01 ~ DQB2*01, DQB1*03 ~ DQB2*01 and DQB1*02 ~ DQB2*01. Thus, we showed that the HD banding patterns obtained using the HURRAH method could be used to distinguish among MHC class II haplotypes and their gene compositions.

**Supplementary Discussion**

Successful analysis of MHC genes requires a great deal of sequence information, including the full-length cDNA, the MHC sequences from at least one individual, and extensive intronic sequences flanking the antigen-presenting exon 2. In a previous study of the giant panda [S4], we investigated exon 2 variations in the *Aime*-DRB1 gene and obtained a 160-bp cDNA fragment of *Aime*-DRB1*02. Only one DRB locus was known at the time, causing us to incorrectly conclude that *Aime*-DRB1 was an expressed gene [S4]. Instead, it is likely that we obtained a mutated clone of *Aime*-DRB3, which was unknown at that time; *Aime*-DRB1*02 and *Aime*-DRB3*07 differ by only one nucleotide within the 160-bp cDNA fragment examined in the previous study. In the present study, in contrast, our HURRAH results suggested that *Aime*-DRB3 is expressed, while *Aime*-DRB1 is a pseudogene, which were also verified by sequence-specific PCR of cDNA (data not shown). In our initial study on *Aime*-DRB3 in the giant panda (unpublished), we repeatedly isolated three cDNA fragments from Jiaozizhizi using exon 2-based 3’RACE: *Aime*-DRB3*01, *Aime*-DRB3*02, and a fragment that we mistakenly called *Aime*-DRB1*01. In fact, the latter proved to be a recombined version of *Aime*-DRB3, once again improperly identified because we were using a partial sequence from exon 2. This highlights the potential for recombination-based errors in studies using RACE-based MHC isolation methods.

MHC genes undergo extensive evolution and purifying selection, which can lead to convergences in the sequences of exon 2 and the other exons. Therefore, RACE-based methods easily produce recombined sequences, especially in cases when researchers seek to amplify a target cDNA sequence that is not expressed, but where a similar sequence may be recombined from an expressed sequence. Furthermore, this kind of recombination problem is a consistent and repeatable issue, meaning that the erroneous MHC sequence could be recovered from other individuals with similar genotypes. This is not an issue in our novel HURRAH protocol, however, as we use hybridization rather than RACE-based PCR to isolate MHC sequences.

**Supplementary Materials and Methods**

Sampling

To validate the efficiency of our new methods, we obtained fresh blood, liver and brain samples from three giant panda individuals, Chenggong (studbook no. 522), Jiaozizhizi (no. 577; death in 2003) and Eryatouzhizi (no. 559; death in 2003), from the Chengdu Breeding Research Base for the Giant Panda. DNA from Chenggong was used for construction of the BAC genomic library [S1]. In addition, we included some BACs from a previous genomic library that had been constructed from another individual, named Xiaojiao (no. 536), and had been employed for MHC assignment [S7]. We used these additional BACs to allow the sequences to be aligned into a full contig with maximum redundancy. Furthermore, 40 giant panda blood samples were collected from the China Research and Conservation Center for the Giant Panda, and used to verify the new protocol.

Isolation of cDNA and genomic sequences

We isolated cDNA sequences according to the standard steps as described in the text. Since partial genomic sequencing of the MHC class II region had already been done for the giant panda [S2], it was not necessary to use LR PCR to isolate the genomic sequences. Instead, we could directly design cg-series primers and use them to amplify BAC controls to verify that the most homozygous SSCP-HD banding patterns could be used to estimate the number of expressed MHC genes. All of the primers involved sequence isolation were listed in Table S2.

Profile reconstitution of SSCP-HD banding patterns at the level of genomic DNA

To reconstitute the genomic DNA-derived SSCP and HD banding patterns of the giant panda, we first used the exon 2-localized cc-series primers (Table S2) to amplify genomic DNA and cDNA samples from Chenggong, Jiaozizhizi, and Eryatouzhizi, as well as from two BACs taken from the giant panda contig ([S1]; Figure S3A). We confirmed the identified loci by aligning the various sequences with the full contig. Using the BAC-based haplotype results revealed by the exon 2-located cc-series primers, we then chose BACs containing multi-locus haplotypes, and used them as controls when validating the HURRAH protocol. We chose 237E1 and 1113F7, which each contained two DQB loci. The DQB cg-series primers were then employed to scan the most homozygous individuals (i.e., those showing the simplest HD patterns).

We first amplified the cg-series PCR products from BAC 237E1, BAC 1113F7, and the genomic DNAs from the most homozygous. We then sequenced six to nine independent amplicons from each. Finally, we used the sequenced clones to reconstitute the SSCP-HD banding patterns derived from BACs and genomic DNA, as described in the text.

**Acknowledgments**

We thank all organizations that provided samples, including the Chengdu Breeding Research Base for the Giant Panda, the China Research and Conservation Center for the Giant Panda.

**References**

1. Zeng CJ, Pan HJ, Gong SB, Yu JQ, Wan QH, Fang SG (2007) Giant panda BAC library construction and assembly of a 650-kb contig spanning major histocompatibility complex class II region. *BMC genomics* 8: 315.
2. Wan QH, Zeng CJ, Ni XW, Pan HJ, Fang SG (2009) Giant panda genomic data provide insight into the birth-and-death process of mammalian major histocompatibility complex class II genes. *PLoS ONE* 4: e4147.
3. Chen Y-Y, Zhang Y-Y, Zhang H-M, Ge Y-F, Wan Q-H, Fang S-G (2010) Natural selection coupled with intragenic recombination shapes diversity patterns in the major histocompatibility complex class ii genes of the giant panda. *J. Exp. Zool. (Mol. Dev. Evol.)* 314B: 208-223.
4. Wan QH, Zhu L, Wu H, Fang SG (2006) Major histocompatibility complex class II variation in the giant panda (*Ailuropoda melanoleuca*). *Mol. Ecol.* 15: 2441-2450.
5. Reche PA, Reinherz EL (2003) Sequence variability analysis of human class I and Class II MHC molecules: functional and structural correlates of amino acid polymorphisms. *J. Mol. Biol.* 331: 623-641.
6. Pan HJ, Wan QH, Fang SG (2008) Molecular characterization of major histocompatibility complex class I genes from the giant panda (*Ailuropoda melanoleuca*). *Immunogenetics* 60: 185-193.
7. Zeng CJ, Yu JQ, Pan HJ. Wan QH, Fang SG (2005) Assignment1 of the giant panda MHC class II gene cluster to chromosome 9q by fluorescence in situ hybridization. *Cytogenet. Genome Res*. 109: 534H.

Supplementary Figure legends

Figure S1. Profile reconstitutes of cDNA-derived SSCP-HD banding patterns based on cc-series PCR products from the giant panda (A: Chenggong; B: Jiaozizhizi; C: Eryatouzhizi), and their use to ensure the completeness of cDNA isolation. The numbers show the electrophoretic lanes, and the letters represent the cDNA sequences isolated. Abbreviations: C, control from conventional cDNA; M, a mix of the products shown in the lanes between C and M.

Figure S2. Multiple sequence alignments of the amino acid sequences deduced from the full-length cDNAs of the MHC class II genes. Dots indicate identity to the first sequence and gaps represent missing amino acids. The box indicates antigen-presenting exon 2, and crosses indicate putative antigen-binding sites, as determined based on the HLA equivalents [S5]. The letters and numbers following the *Aime*-MHC genes indicate their corresponding cDNA sequences, and the names of loci and alleles identified by the HURRAH protocol.

Figure S3. Genomic DNA-derived SSCP-HD banding patterns reconstituted using cg-series PCR products amplified from the giant panda (A-C). BACs from a full and maximally redundant contig assembled from two individuals were used as controls (A). BACs shown in black were derived from Chenggong, while those in gray were from Xiaojiao. The letters a, b, c and d represent the cDNA sequences. *Only *Aime*-DRB3 is expressed; the other two DRB loci are pseudogenes (gray). **DQA2 is tissue-specifically expressed, as indicated by the absence of DQA2*02 in mRNA derived from Chenggong’s blood. Here, we showed reconstitution of two homozygous multi-DQB haplotypes; DQB1*04~DQB2*01 from BACs 237E1 and 1113F7 (B) and DQB1*03~DQB2*01 from the G individual (C). We successfully reconstituted the standard BAC-based multi-DQB haplotype (B), and a homozygous multi-DQB haplotype identified from SSCP-HD examination of 40 giant pandas (C). Abbreviations: G, control from genomic DNA; M, a mix of the products shown in the lanes between BAC/G and M.

Table S1. Genotypic data for the giant panda individuals used for profile reconstitution of the cDNA-derived SSCP-HD banding patterns.

| Individual  (studbook #, cDNA source) | | DRA | DRB1 | DRB2 ѱ | DRB3 | DQA1 | DQB1 | DQA2 | DQB2 |
| --- | --- | --- | --- | --- | --- | --- | --- | --- | --- |
| Chenggong (No. 522, blood) | genomic genotype ^a^ | 1*01 | 1*01 | 2*01 | 3*01 | 1*02 1*03 | 1*01 1*04 | 2*01 2*02 | 2*01 |
|  | cDNA hybridization | 1*01a | / | / | 3*01a 1*02'b | 1*02a 1*03b | 1*01a 1*04b | / | 2*01d |
|  | cc-primer validation | 1*01a | / | / | 3*01a 1*02'b | 1*02a 1*03b | 1*01a 1*04b | / | 2*01d |
|  | corrected genotype | 1*01 | 1*01 | 2*01 | 3*01 3*07 | 1*02 1*03 | 1*01 1*04 | 2*01 2*02 | 2*01 |
| Jiaozizhizi (No. 577, liver) | genomic genotype ^a^ | 1*01 | 1*01 | 2*01 | 3*01 3*02 | 1*02 1*03 | 1*02 1*04 | 2*01 2*02 | 2*01 |
|  | cDNA hybridization | 1*01a | / | / | 3*01a 3*02c | 1*02a 1*03b | 1*02c 1*04b | / | 2*01d |
|  | cc-primer validation | 1*01a | / | / | 3*01a 3*02c | 1*02a 1*03b | 1*02c 1*04b | / | 2*01d |
|  | corrected genotype | 1*01 | 1*01 | 2*01 | 3*01 3*02 | 1*01 1*03 | 1*02 1*04 | 2*01 2*02 | 2*01 |
| Eryatouzhizi (No. 559, brain) | genomic genotype ^a^ | 1*01 | 1*02 1*03 | 2*01 | 3*01 | 1*03 1*05 | 1*02 1*04 | 2*01 | 2*01 |
|  | cDNA hybridization | 1*01a | / | / | 3*01a 1*01'd | 1*03b 1*05c | 1*02c 1*04b | 2*01d | 2*01d |
|  | cc-primer validation | 1*01a | / | / | 3*01a 1*01'd | 1*03b 1*05c | 1*02c 1*04b | 2*01d | 2*01d |
|  | corrected genotype | 1*01 | 1*02 1*03 | 2*01 | 3*01 3*08 | 1*03 1*05 | 1*02 1*04 | 2*01 | 2*01 |
| Xiaojiao ^b^ (No. 536) | genomic genotype ^a^ | 1*01 | 1*01 1*03 | 2*01 | 3*02 | 1*02 1*05 | 1*04 | 2*01 | 2*01 |
|  | corrected genotype | 1*01 | 1*01 1*03 | 2*01 | 3*02 3*08 | 1*02 1*05 | 1*04 | 2*01 | 2*01 |
|  | Confirmation of  gene function | Expressed | Pseudo | Pseudo | Expressed | Expressed | Expressed | Specifically expressed | Expressed |

^a^ The expected genotypes of these individuals were derived from Chen et al. [S3] and Wan et al. [S4].

^b^ We failed to obtain fresh blood or tissue samples from Xiaojiao for mRNA extraction.

Table S2. Common cDNA (cc-series) and genomic DNA (cg-series) primers for the giant panda; these were used to confirm that all relevant sequences had been isolated, as assessed by reconstitution of SSCP-HD profiles. We designed two cc-primer pairs (cc1 and cc2); cc1 spanned exons 1~3, while the nested cc2 was located in exon 2 and could be used to compare the exon 2-based SSCP-HD profiles obtained from cDNA and BAC templates.

| Locus | Name | Primer sequence (5’ → 3’) | Ta (°C) | Size (bp) |
| --- | --- | --- | --- | --- |
| DRA | DRAcc1 | F: GTCCCCAGGAATCACAGGCTATC | 57.0 | 317 |
|  |  | R: CAGTTCCACAGGGGTGTTTGAGAG |  |  |
|  | DRAcc2 | F: AGGACCATGTGATCATCCAGGC | 57.0 | 245 |
|  |  | R: ATTGGTGTTCGGGGTGTGGTTG |  |  |
| DRB | DRBcc1 | F: TGGCATGGGCTAGGGACACC | 61.5 | 338 |
|  |  | R: CAGGGGCCGGGTCTTCGTA |  |  |
|  | DRBcc2 | F: CGAGTGCTACTTCACCAACGG | 60.0 | 244 |
|  |  | R: TTGTCGCTGCACCAGGAAGCT |  |  |
| DQA | DQAcc1 | F: CATTGTGGCTGACCATGTTGC | 56.0 | 305 |
|  |  | R: GACCCAGCATCACAGGAGACTTG |  |  |
|  | DQAcc2 | F: ATGGCATAAATGTCTACCAGTCTTA | 53.0 | 231 |
|  |  | R: CATTGGTAGCAGCGGTATAGTTGGA |  |  |
| DQB | DQBcc1 | F: GAGCGTCCCAGTGGCTGAGG | 61.0 | 338 |
|  |  | R: TCCTGGATGGGGAGATGGTCAC |  |  |
|  | DQBcc2 | F: TTCCAGTTTAAGGGCGAGTGCTA | 60.0 | 256 |
|  |  | R: GCCGCTGCAGGATGAACC |  |  |
|  | DQBcg | F: GTTCATCAGGCCCCTGGTAGTGCT | 62.5 | 390 |
|  |  | R: CGAACGGCCTGGCTCACCTC |  |  |


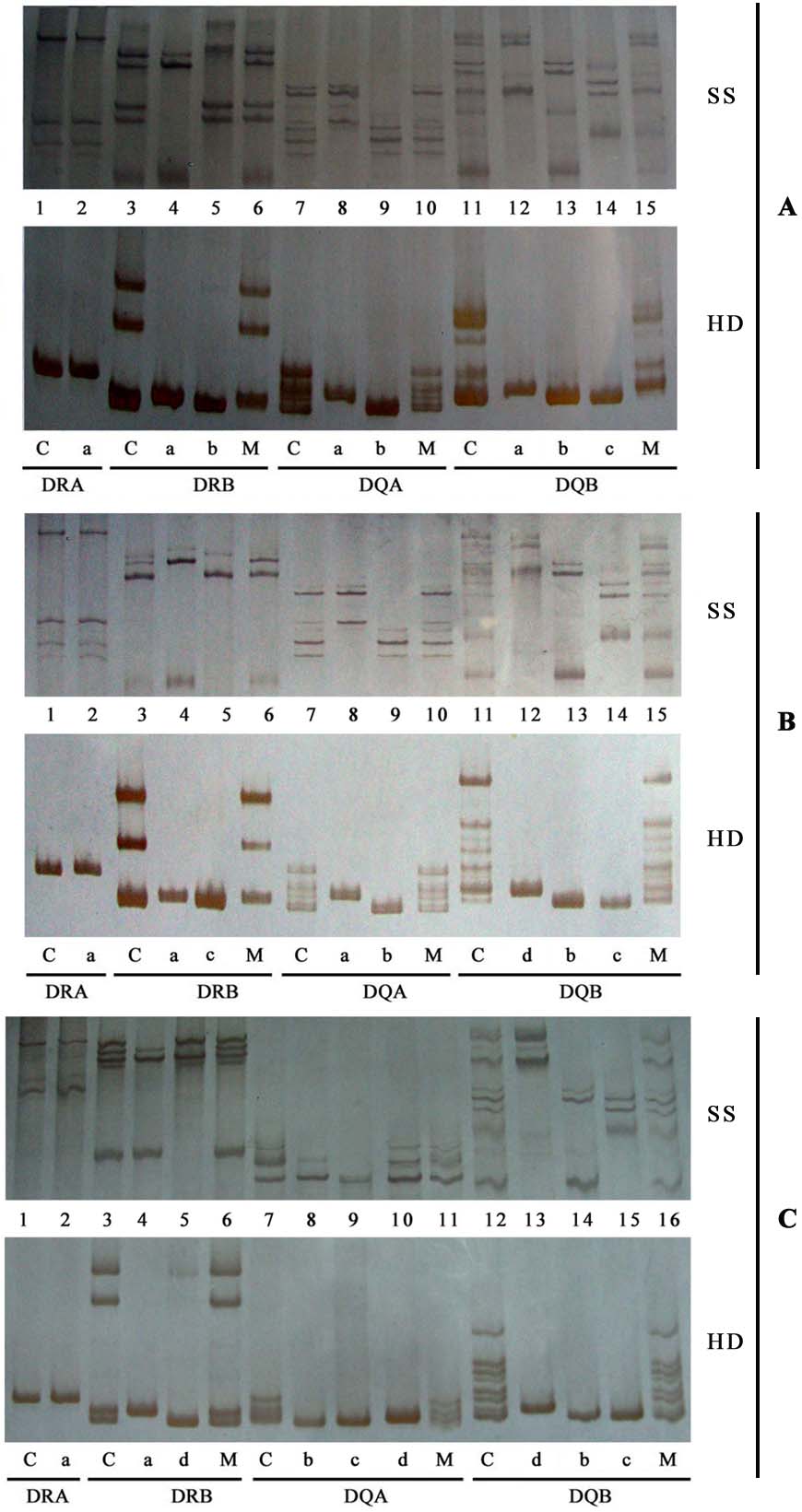


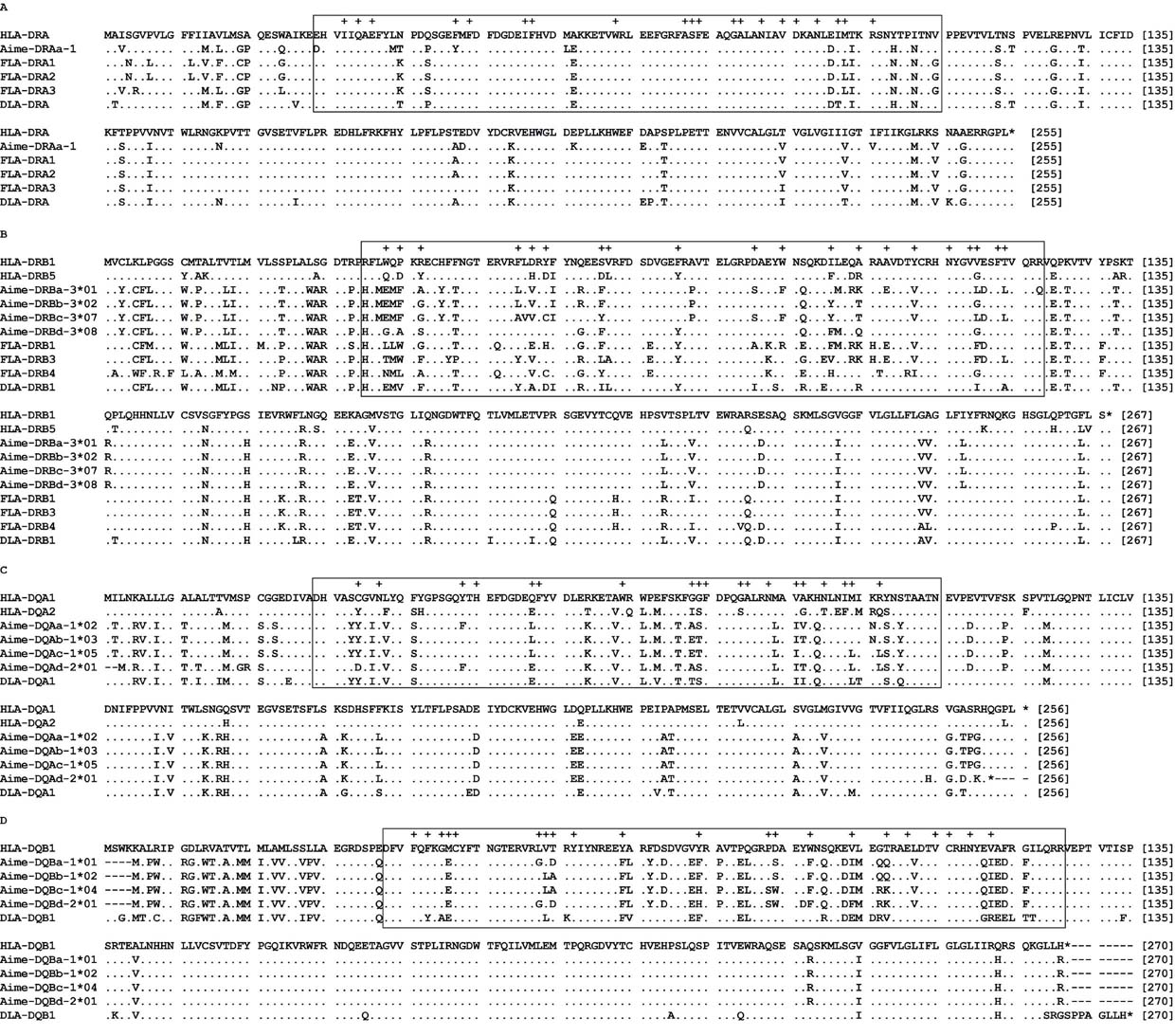


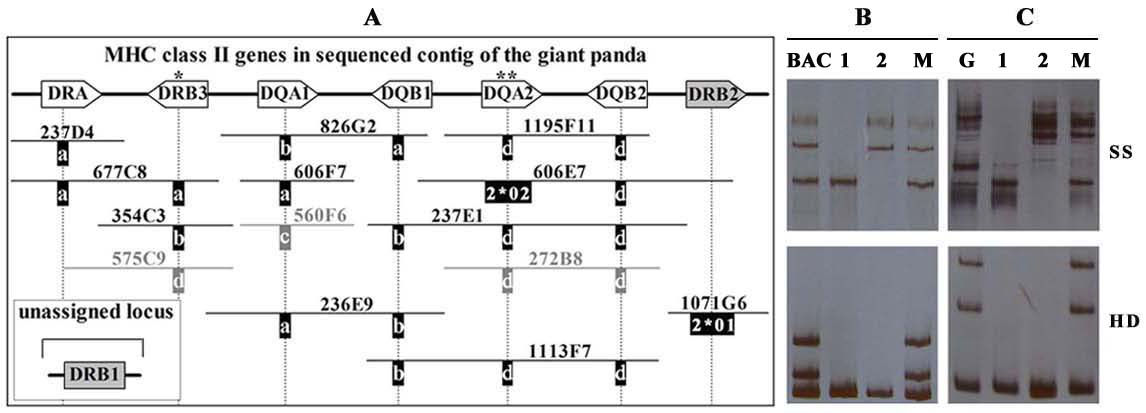

Supplement: Data S1 — Verification of the novel protocol using the giant panda. (0.57 MB DOCX) [file pone.0014518.s001.docx]
