# Supplementary figures and images for: A Novel HURRAH Protocol Reveals High Numbers of Monomorphic MHC Class II Loci and Two Asymmetric Multi-Locus Haplotypes in the Père David's Deer
Source: PLoS One. 2011 Jan 18;6(1):e14518. doi: 10.1371/journal.pone.0014518 (PMC3022581; doi:10.1371/journal.pone.0014518)

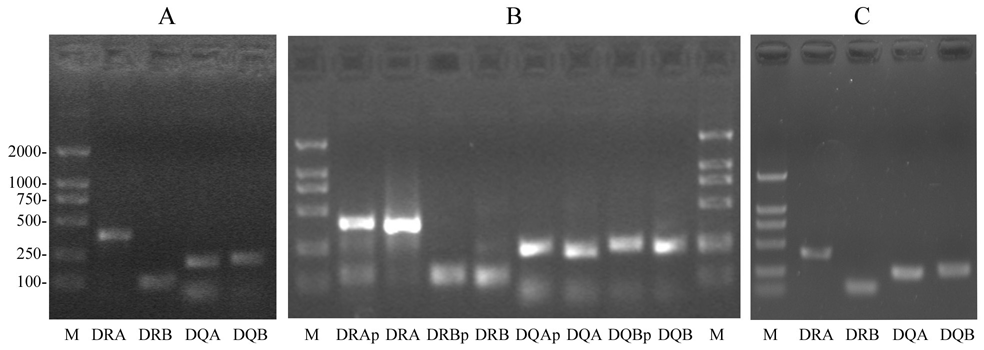

Supplement: Figure S1 — Conventional amplification and biotinylated probe preparation using u-series universal primers. A) PCR products from the giant panda. B) Comparison between normal PCR products and biotinylated probes (marked as DRAp, DRBp, DQAp, and DQBp). C) PCR products from the Père David's deer. (0.15 MB TIF) [file pone.0014518.s002.tif]

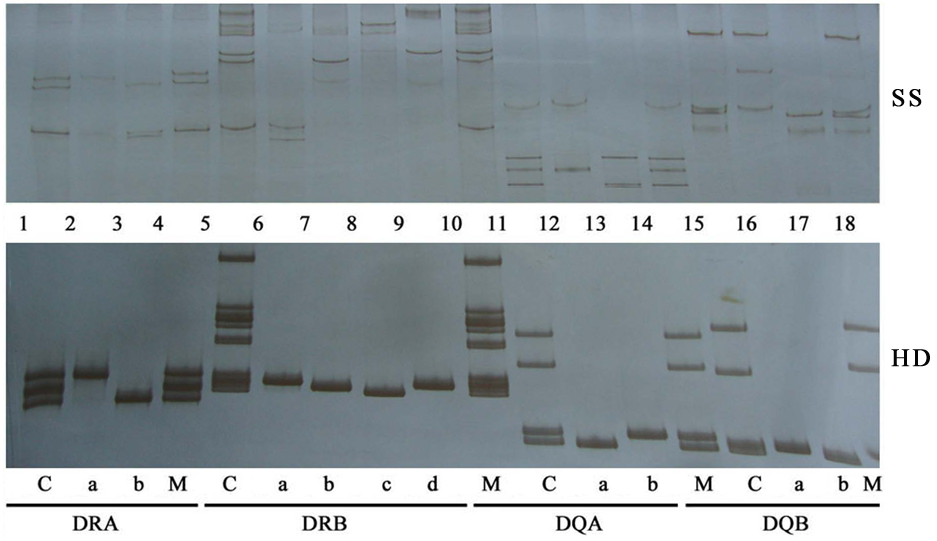

Supplement: Figure S2 — Profile reconstitutes of cDNA-derived SSCP-HD banding patterns based on cc-series PCR products from the Père David's deer, and their use to ensure the completeness of cDNA isolation. The numbers show the electrophoretic lanes, and the letters represent the cDNA sequences isolated. Abbreviations: C, control from conventional cDNA; M, a mix of the products shown in the lanes between C and M. (0.41 MB TIF) [file pone.0014518.s003.tif]

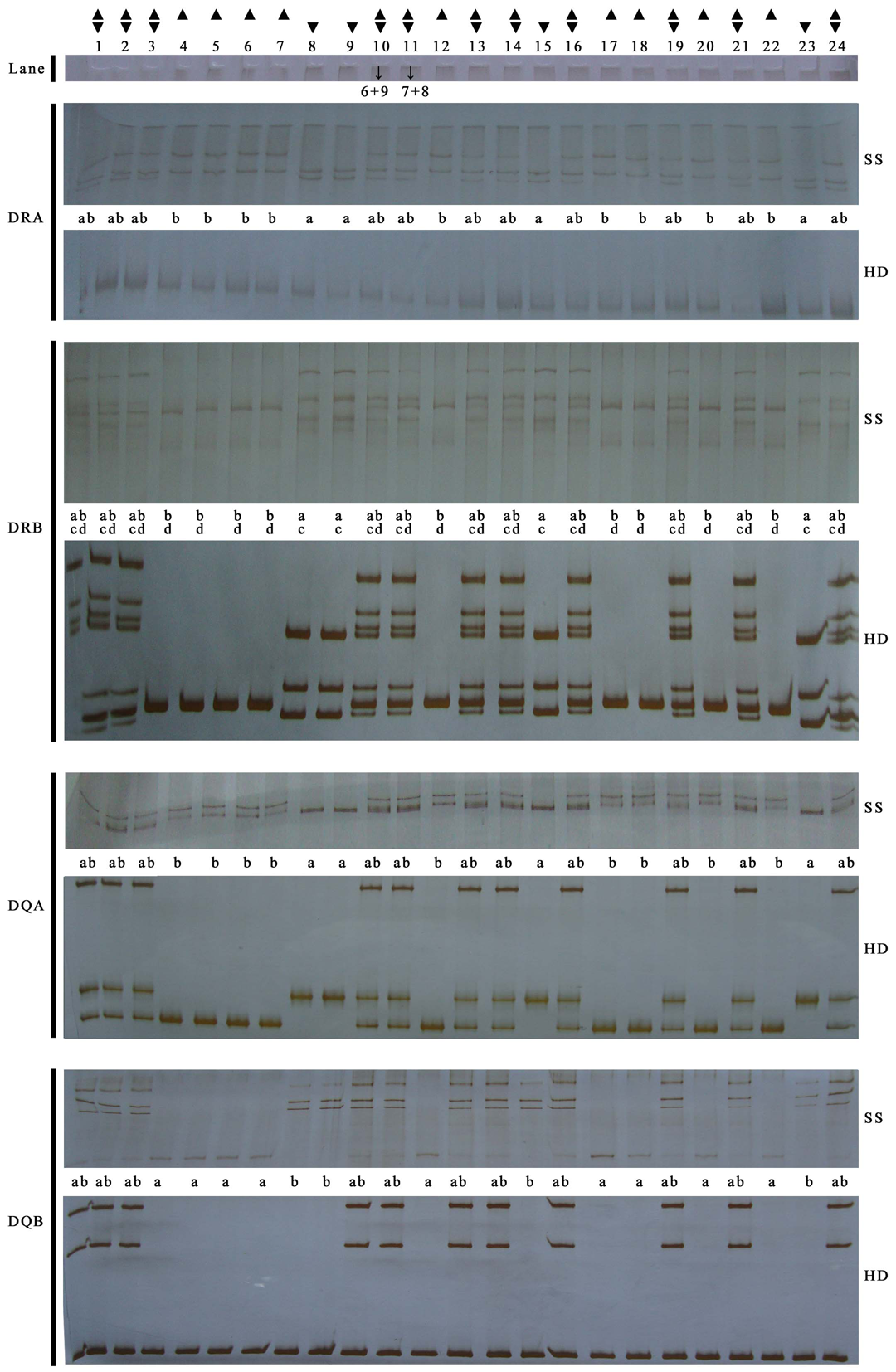

Supplement: Figure S3 — The SSCP-HD banding patterns for the cg-series PCR products of the Père David's deer. The same individuals were investigated for the DRA, DRB, DQA, and DQB genes. The banding patterns designated with letters a, b, c, d correspond to the cDNA sequences isolated. The symbols ▴, ▾, and ▴+▾ indicate the homozygous H1, homozygous H2 and heterozygous H1/H2 haplotypes, respectively. Here, we also show two mixed samples (lane 10 is a mixture of lanes 6 and 9, and lane 11 is a mixture of lanes 7 and 8) that reconstitute the H1/H2 heterozygote. (1.53 MB TIF) [file pone.0014518.s004.tif]

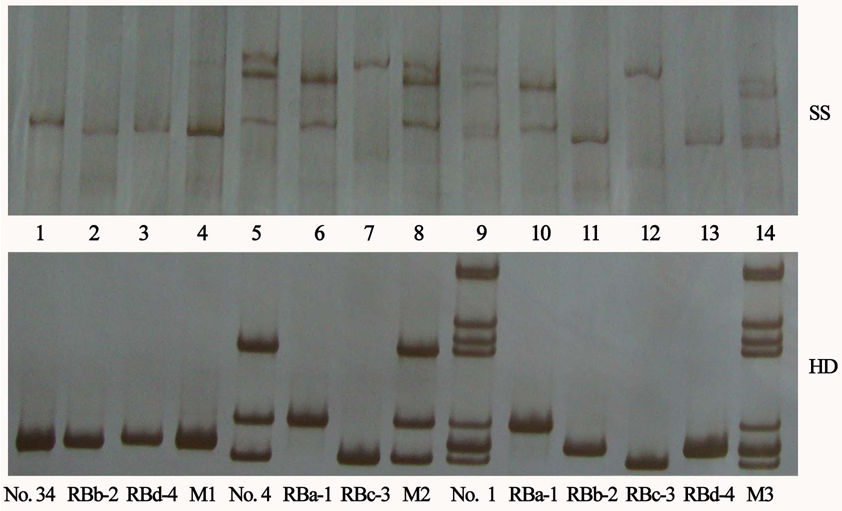

Supplement: Figure S4 — Genomic DNA-derived SSCP-HD banding patterns reconstituted using cg-series PCR products amplified from the Père David's deer. The homozygous DQA and DQB haplotypes genes each only represented one locus, so we chose to reconstitute the two homozygotes and one heterozygote of the Elda-DRB haplotype. Ear tag numbers 34, 4 and 1 are deer that showed the H1, H2 and H1/H2 DRB haplotypes, respectively. The information beside each Elda-MHC gene indicates the initial cDNA sequences (a, b, c and d) and the subsequently identified loci (1, 2, 3 and 4). (0.50 MB TIF) [file pone.0014518.s005.tif]
