# Supplementary material for: A Novel HURRAH Protocol Reveals High Numbers of Monomorphic MHC Class II Loci and Two Asymmetric Multi-Locus Haplotypes in the Père David's Deer
Source: PLoS One. 2011 Jan 18;6(1):e14518. doi: 10.1371/journal.pone.0014518 (PMC3022581; doi:10.1371/journal.pone.0014518)
Supplement: Table S1 — Universal (u-series) primers used for probe preparation, which successfully amplified DNA from the carnivorous giant panda and the herbivorous Père David's deer. (0.04 MB DOC) [file pone.0014518.s006.doc]

Table S1. Universal (u-series) primers used for probe preparation, which successfully amplified DNA from the carnivorous giant panda and the herbivorous Père David’s deer*.

| Locus | Name | Primer sequence (5’ → 3’) | Ta (°C) | Size (bp) |
| --- | --- | --- | --- | --- |
| DRA | DRAu | F: CA/C/TAC/TACC/TCCA/GAA/TCACCAATG | 57.5 | 387 |
|  |  | R: ATGATGCCCACCAGAG/CCCAC |  |  |
| DRB | DRBu | F: CCTGCAGCACCACAACCT | 53.5 | 103 |
|  |  | R: CACCCCAGC/TCTC/TCTCTTC |  |  |
| DQA | DQAu | F: CAGCCCAACACCCTCATCTG | 56.0 | 212 |
|  |  | R: TCCAGGCCCCAGTGCTCC |  |  |
| DQB | DQBu | F: TCTGC/TT/GCG/AGTGACAGATTTCT | 60.0 | 227 |
|  |  | R: TGA GCC CGC CAC TCC AC |  |  |

*The following sequences were used to design the u-series primers: **for the** **DRAs:** AB008754, AB215118-9, AJ586874-84, AK231578, AK239665, AY191779, AY243102, AY243106, AY247781-3, AY285926, AY285928-9, AY285933, AY285935-9, AY459302, AY491450, AY491451-5, BC032350, BC071659, BC102053, CR457013, CR590143, CR594558, CR613451, D37955-6, DQ016629, DQ298395, DQ883222-4, DQ891595, DQ894789, J00194, K01171, L27739, L37332, M60334, M73983, M92445, M93028, NM_001011723, NM_001012677, NM_019111, U51576, U51578, X78308, XM_518384; **for the** **DRBs:** AB008345-6, AF192258, AF291675, AF336340-1, AF387317, AF492273, AF492278, AF492280, AF492285, AF492288, AF492295, AF492300, AJ245881, AJ297586, AJ536121, AJ556173, AJ601349, AJ601354, AJ601356, AJ601359, AJ601362, AJ601366-7, AM180935-8, AM182980-2, AY125892-3, AY626552, AY770514, AY961062-3, AY961068, AY961072, AY961074-5, AY961078, BC001023, BC024269, BC033827, BC102875, BC108922, BT025473, D45357-9, DQ002917, DQ057985, DQ146947, DQ223553, DQ284432, DQ284435, DQ381747, DQ381755, L04790, L27740, L42143, M15992, M16956-7, M17378, M17381, M20430, M20503, M21008, M28584, M32578, M73984, M77154, M93432, NM_001012680, NM_001040030, NM_002124, S40615, U84892, X02902, X12544, X92409, X99896, XM_001165293, XM_001165330, XM_001165397, XM_001165464, XM_001165493-4, XM_001165686, XM_001165717, XM_001165744, XM_001165773, XM_001165809, XM_518387, XM_582099, Y18246, Y18308; **for the** **DQAs:** AB209628, AB215111-7, AF502560-4, AK130598, AK130811, AK130838, AK230483, AK232063, AK236590, AK239488, AK240184, AY102474-5, AY191777, AY243100, AY243104, AY247776-7, AY285925, AY285930, AY285932, AY285934, AY459303, AY464652, AY730727, AY879112, AY906855, AY954685, BC008585, BC073977, BC102345, BC102953, BC112472, BC125044-5, BT020994, CR450297, CR859909, D50045, D50047, D50049, D50454, DQ003300, DQ275670, DQ284436, DQ284437-40, DQ440647-8, DQ883217, DQ891535, DQ894724, DQ917643, M16995, M26041, M29939, M33906, M93430-1, M93433, NM_001012675, NM_001012681, NM_001013601, NM_002122, NM_020056, U77589, X00033, X00370, X00452, XM_001115365, XM_001129369, XM_001165913, XM_527352, XM_527354, XM_936128, XM_936213, Y07819-20, Y07898, Y14020-2; **and for the DQBs:** AB010577, AF195245, AF464026-7, AF464029-30, AF464038, AF464048, AF503398, AF503401, AF503406-7, AK098007, AK232574, AY135568, AY135574, AY247778-80, AY459301, AY656682, AY656683, AY730728, AY911331, BC012106, BC118130, CR615403, CR858208, D37952-4, DQ892298, DQ895497, J00198, L08792, L33910, L34095-107, L40179-80, L42626, M16996, M17563-65, M17955, M20432, M20505, M26042, M31498, M32120, M32577, M33907, M59826, M60028, M81140-1, NM_001012676, NM_001034668, NM_002123, U83582, X00369, X03068, X96420, XM_001164870, XM_001164906, XM_001164946, XM_001166263, XM_001166297, XM_518388, XR_013474, Y18201, Y18202, Y18203.
