# Supplementary material for: A Novel HURRAH Protocol Reveals High Numbers of Monomorphic MHC Class II Loci and Two Asymmetric Multi-Locus Haplotypes in the Père David's Deer
Source: PLoS One. 2011 Jan 18;6(1):e14518. doi: 10.1371/journal.pone.0014518 (PMC3022581; doi:10.1371/journal.pone.0014518)
Supplement: Table S2 — Common cDNA (cc-series) and genomic DNA (cg-series) primers for the Père David's deer; these were used to confirm that all relevant sequences had been isolated, as assessed by reconstitution of SSCP-HD profiles. (0.04 MB DOC) [file pone.0014518.s007.doc]

Table S2. Common cDNA (cc-series) and genomic DNA (cg-series) primers for the Père David’s deer; these were used to confirm that all relevant sequences had been isolated, as assessed by reconstitution of SSCP-HD profiles.

| Locus | Name | Primer sequence (5’ → 3’) | Ta (°C) | Size (bp) |
| --- | --- | --- | --- | --- |
| DRA | DRAcc | F: GATGGGCCTCTTCTCAAGCACT | 56.0 | 354 |
|  |  | R: TGGAGACTATGGAAGGCATCTTAC |  |  |
|  | DRAcg | F: CAACTCATCCCTACTCTCCATCAC | 56.5 | 487 |
|  |  | R: AGGCGAAAGTCTCAGTAACAAAAA |  |  |
| DRB | DRBcc | F: GTGCCTGTATTTCTCTGGAGGCTC | 60.5 | 411 |
|  |  | R: TGCAGGGGCTGGGTCTTTG |  |  |
|  | DRBcg | F: TAGTTGGGGTGCCGGTGGAG | 61.0 | 433 |
|  |  | R: TCGCCGCTGCACAGTGAAACT |  |  |
| DQA | DQAcc | F: CTCGCCCTGACCACCATGATGA | 57.0 | 299 |
|  |  | R: TTGTTGGTAGCAGCGGTAGAGTTGG |  |  |
|  | DQAcg | F: CAAGGCTTTTTAACTAACTGGACAACTA | 55.0 | 457 |
|  |  | R: AGAGGCAGAATGGTGAACACATAC |  |  |
| DQB | DQBcc | F: CCAGGGGCTGAGGGCAGAGAC | 62.0 | 337 |
|  |  | R: CCTCAGTCCTGGACGGGGAGAT |  |  |
|  | DQBcg | F: AGAGGTGGCGGGTTTCAGGTTTA | 61.0 | 352 |
|  |  | R: GTAGTTGTGTCTGCACACCGTGTC |  |  |
