# Supplementary material for: A Novel HURRAH Protocol Reveals High Numbers of Monomorphic MHC Class II Loci and Two Asymmetric Multi-Locus Haplotypes in the Père David's Deer
Source: PLoS One. 2011 Jan 18;6(1):e14518. doi: 10.1371/journal.pone.0014518 (PMC3022581; doi:10.1371/journal.pone.0014518)
Supplement: Table S3 — Exon-specific (es-series) primers for the Père David's deer were used to isolate the genomic introns of each isolated cDNA sequence. All primer pairs were subjected to LR PCR, using 68°C as the annealing and extension temperature. (0.05 MB DOC) [file pone.0014518.s008.doc]

Table S3. Exon-specific (es-series) primers for the Père David’s deer were used to isolate the genomic introns of each isolated cDNA sequence. All primer pairs were subjected to LR PCR, using 68°C as the annealing and extension temperature.

| Locus | Name | Primer sequence (5’ → 3’) | Ta (°C) | Size |
| --- | --- | --- | --- | --- |
| DRAa-1*01 | gRAa E1-E4 | F: CCTACAGGAATCAGGGGCTATCAA | 68.0 | 3.6 kb |
|  |  | R: TTACCTGCAGGTGCCTCAGAGAG |  |  |
| DRAb-1*02 | gRAb E1-E4 | F: CCTACAGGAATCAGGGGCTATCAA | 68.0 | 3.6 kb |
|  |  | R: TTACCTGCAGGGGCCTCACAGT |  |  |
| DRBa-1 | gRB1 E1-E2 | F: CCTGCTCCTCTCACTCTCCTC | 68.0 | 7.9 kb |
|  |  | R: AGCGCACCAACTCTTCTCC |  |  |
|  | gRB1 E2-E3 | F: GAGTGTCATTTCTCCAACGGGAC | 68.0 | 2.5 kb |
|  |  | R: ACCATTCACGGAGCAGACCAG |  |  |
| DRBb-2 | gRB2 E1-E2 | F: CTTGCCTGCTCCTCTCACTCTCTG | 68.0 | 6.6 kb |
|  |  | R: TCCACCTCGGCCCGGTG |  |  |
|  | gRB2 E2-E3 | F: CACATTTCCTGGAGCATATTAAGG | 68.0 | 2.4 kb |
|  |  | R: CACGGTCACTGTAGGCTCCACTCG |  |  |
| DRBc-3 | gRB3 E1-E2 | F: CTTGCCTGCTCCTCTCACTCTCTG | 68.0 | 6.4 kb |
|  |  | R: GGAGAAATGACACTCGCTCTTAGC |  |  |
|  | gRB3 E2-E3 | F: CCAGAAGGAGTTCCTGGAGCAGAG | 68.0 | 2.3 kb |
|  |  | R: GGGTCTTTGCAGGATACACGGTCACT |  |  |
| DRBd-4 | gRB4 E1-E2 | F: GAGGCTCCTGGATGGCAGCTCTAACA | 68.0 | 7.3 kb |
|  |  | R: GCCGCTGCACAGTGAAACTCTCAATA |  |  |
|  | gRB4 E2-E3 | F: CTGCAGACACAACTACGGGGTTAT | 68.0 | 2.5 kb |
|  |  | R: TACTGTGATAGGGCTCGTCTGGCC |  |  |
| DQAa-1 | gQA1 E1-E3 | F: GCCCTGACCACCATGATG | 68.0 | 3.0 kb |
|  |  | R: AGGGAGGAAGGTGAGGTAACTG |  |  |
| DQAb-2 | gQA2 E1-E3 | F: GCCCTGACCACCATGATG | 68.0 | 3.9 kb |
|  |  | R: GGTCTCAGAAACACCCTCTGTG |  |  |
| DQBa-1 | gQB1 E1-E3 | F: ATTATGTCTGGGATGGTGGCTCTG | 68.0 | 6.2 kb |
|  |  | R: GGGGGTCATCTCCAACATCACAAGT |  |  |
| DQBb-2 | gQB2 E1-E3 | F: ATTATGTCTGGGATGGTGGCTCTG | 68.0 | 6.0 kb |
|  |  | R: CAGCATCACGAGGATCTGGAAGC |  |  |
